# The universal Sua5/TsaC family evolved different mechanisms for the synthesis of a key tRNA modification

**DOI:** 10.3389/fmicb.2023.1204045

**Published:** 2023-06-21

**Authors:** Adeline Pichard-Kostuch, Violette Da Cunha, Jacques Oberto, Ludovic Sauguet, Tamara Basta

**Affiliations:** ^1^CEA, CNRS, Institute for Integrative Biology of the Cell (I2BC), Université Paris-Saclay, Gif-sur-Yvette, France; ^2^Architecture and Dynamics of Biological Macromolecules, Institut Pasteur, Université Paris Cité, CNRS, UMR 3528, Paris, France

**Keywords:** universal proteins, enzyme, Sua5, TsaC, t^6^A, tRNA, evolution

## Abstract

TsaC/Sua5 family of enzymes catalyzes the first step in the synthesis of N6-threonyl-carbamoyl adenosine (t^6^A) one of few truly ubiquitous tRNA modifications important for translation accuracy. TsaC is a single domain protein while Sua5 proteins contains a TsaC-like domain and an additional SUA5 domain of unknown function. The emergence of these two proteins and their respective mechanisms for t^6^A synthesis remain poorly understood. Here, we performed phylogenetic and comparative sequence and structure analysis of TsaC and Sua5 proteins. We confirm that this family is ubiquitous but the co-occurrence of both variants in the same organism is rare and unstable. We further find that obligate symbionts are the only organisms lacking *sua5* or *tsaC* genes. The data suggest that Sua5 was the ancestral version of the enzyme while TsaC arose via loss of the SUA5 domain that occurred multiple times in course of evolution. Multiple losses of one of the two variants in combination with horizontal gene transfers along a large range of phylogenetic distances explains the present day patchy distribution of Sua5 and TsaC. The loss of the SUA5 domain triggered adaptive mutations affecting the substrate binding in TsaC proteins. Finally, we identified atypical Sua5 proteins in Archaeoglobi archaea that seem to be in the process of losing the SUA5 domain through progressive gene erosion. Together, our study uncovers the evolutionary path for emergence of these homologous isofunctional enzymes and lays the groundwork for future experimental studies on the function of TsaC/Sua5 proteins in maintaining faithful translation.

## Introduction

**Figure 1:**
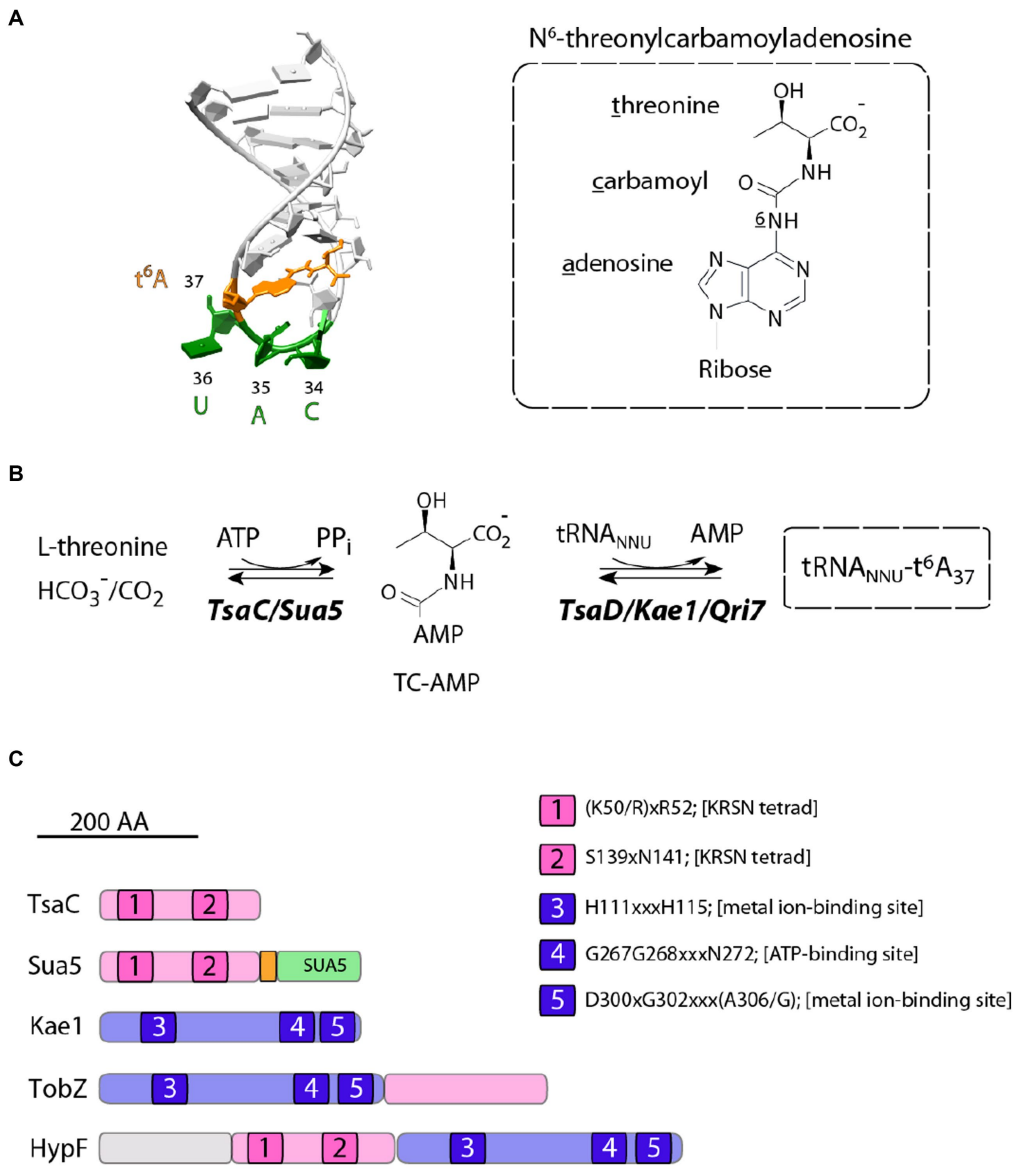
Structure and biosynthesis of t^6^A_37_ nucleoside. **(A)** On the left is shown the structure of the anticodon loop of Schizosaccharomyces pombe tRNA^iMet^ (PDB 2G1G). The anticodon residues and t^6^A_37_ nucleoside are colored in green and orange, respectively. The chemical formula of t^6^A_37_ is depicted on the right. **(B)** Proposed mechanism for t^6^A_37_ biosynthesis. **(C)** Primary structures of proteins containing TsaC orthologous domains. All proteins are drawn to scale and the approximate position of conserved motifs (1–5) is indicated. The numbering corresponds to that of *E. coli* TsaC and TsaD proteins. Sua5 proteins contain TsaC-orthologous domain with a loop of about 20 residues (orange) fused to SUA5 domain of about 100 residues (green). TobZ proteins catalyse the formation of the antibiotic nebramycin using the hydrolysis of carbamoyl phosphate and its subsequent adenylation by ATP to yield O-carbamoyladenylate. HypF is a carbamoyl transferase involved in the maturation of [NiFe] hydrogenases. HypF uses carbamoylphosphate as a substrate and transfers the carboxamido moiety in an ATP-dependent reaction to the thiolate of the C-terminal cysteine of HypE yielding a protein-S-carboxamide.

tRNA requires post-transcriptional maturation to be functional during the translation process. This involves *inter alia* the enzymatic modification of the canonical A, U, C and G bases to form modified nucleosides by addition of a variety of chemical groups (Väre et al., 2017). About hundred different modified nucleosides have been identified so far in tRNA, 20 of which are universal and were likely inherited from the Last Universal Common Ancestor (LUCA; Cantara et al., 2011; Machnicka et al., 2014). Among those, N^6^-threonyl-carbamoyl-adenosine (t^6^A) is found on the adenosine in position 37, next to the anticodon of almost all tRNAs that recognize ANN codons (where N = G, A, C or U; Figure 1A; Ishikura et al., 1969; Powers and Peterkofsky, 1972).

t^6^A is required for translation accuracy (Högenauer et al., 1972; Weissenbach and Grosjean, 1981; Stuart et al., 2000; Murphy et al., 2004; El Yacoubi et al., 2011), aminoacylation of some tRNA species (Nureki et al., 1994), translation initiation (Thiaville et al., 2016; Llácer et al., 2018) and translocation of the ribosome on mRNA (Phelps et al., 2004). The mutations in t^6^A synthetic genes were linked with a variety of phenotypes in eukaryotes and prokaryotes ranging from cell death or slow growth to short telomeres and defects in transcription probably reflecting the far-reaching indirect consequences of the unfaithful translation (El Yacoubi et al., 2009; Naor et al., 2012; Beenstock and Sicheri, 2021). In humans, mutations in all t^6^A-synthetic genes were linked with Galloway-Mowat syndrome a severe genetic disease causing brain malformation and renal dysfunction (Braun et al., 2017; Edvardson et al., 2017; Arrondel et al., 2019).

t^6^A-tRNA biosynthesis proceeds in two steps and requires multiple proteins. The universal enzyme family Sua5/TsaC uses L-threonine, ATP and bicarbonate/carbon dioxide (HC0_3_^−^/CO_2_) to synthesize the activated intermediate threonyl-carbamoyl-adenylate (TC-AMP) via a complex and poorly understood mechanism (Figure 1B; El Yacoubi et al., 2009; Lauhon, 2012; Perrochia et al., 2013a,b; Harris et al., 2015). In the second step, the threonyl-carbamoyl moiety is transferred from the TC-AMP onto the substrate tRNA. This reaction is catalyzed by Kae1/TsaD/Qri7 universal protein family (Figure 1B) which associates with accessory proteins to form the DEZ complex in bacteria and the KEOPS complex in archaea and eukaryotes [reviewed in Thiaville et al. (2014b)].

TsaC/Sua5 family of proteins is a part of a restricted set of about 60 proteins shared by all cellular organisms (Koonin, 2003; Galperin, 2004). Remarkably, this family is composed of two distinct variants, TsaC and Sua5, which share a homologous catalytic domain but differ in the presence of a second domain, named SUA5 (Figure 1C), found only in Sua5 proteins. Initial bioinformatics analysis reported no clear phyletic pattern underpinning the distribution of TsaC and Sua5 proteins and additionally showed that the simultaneous occurrence of both genes in a genome seems to be rare (Thiaville et al., 2014b). TsaC-like domain is also found fused to Kae1-like domain in HypF proteins involved in in the maturation of [NiFe] hydrogenases and in TobZ proteins that synthetize the antibiotic nebramycin (Figure 1C). In these proteins, the TsaC-like domains use carbamoyl phosphate to catalyze a carbamoylation reaction similar to a putative step in TC-AMP synthesis (Petkun et al., 2011; Parthier et al., 2012).

The structures of TsaC from *E. coli* (*Ec-*TsaC; Teplova et al., 2000; Harris et al., 2015) and Sua5 from the *Sulfolobus tokodaii* (*St*-Sua5; Agari et al., 2008; Kuratani et al., 2011; Parthier et al., 2012) and *Pyrococcus abyssi* (*Pa*-Sua5; Pichard-Kostuch et al., 2018) show that the catalytic domain adopts a globular twisted fold made of 7 parallel and anti-parallel β-strands bordered by 7 α-helices. The active site is in the cavity formed by this domain and contains highly conserved residues K^56^xR^58^/S^143^xN^145^ and T^33^/S^181^/R^195^ (*Pa*-Sua5 numbering) involved in ATP and threonine binding, respectively. The mutational analyses showed that these residues were all required for TC-AMP synthesis by Sua5 from *S. cerevisiae* (Wan et al., 2013).

The TsaC-like catalytic domain of Sua5 proteins carries a C-terminal extension of about 20 residues forming a flexible loop that contains a set of highly conserved residues, Pro^228^-Gly^229^-Met^230^ and His^234^-Tyr^235^ (*Pa*-Sua5 numbering; Pichard-Kostuch et al., 2018). The loop folds into the active site gorge and was suggested to act as a gate regulating the binding and release of ligands (Pichard-Kostuch et al., 2018).

The C-terminal extremity of the loop is anchored in the SUA5 domain which is composed of about 100 residues (Agari et al., 2008; Kuratani et al., 2011; Pichard-Kostuch et al., 2018). The domain exhibits an atypical Rossman fold composed of five β-strands (β12-β16) and three α-helices (α9-α11). SUA5 domain and the catalytic TsaC-like domain form a tight interface that was shown to be important for the activity (Pichard-Kostuch et al., 2018).

How TsaC and Sua5 emerged in course of evolution and what are the functional differences between these two different protein scaffolds remains elusive. To address these questions, we performed a comprehensive phylogenetic, structural, and sequence analysis of Sua5/TsaC proteins. The data suggest pre-LUCA origin of this family and a complex evolution including gene erosion, multiple gene losses and horizontal gene transfers. We identified conserved residues specific for TsaC or Sua5 proteins and tentatively assigned them a role in substrate binding. Finally, we identified atypical Sua5 proteins which seem to be in the process of losing the SUA5 domain thus potentially ongoing the transition toward TsaC homologs. Together, this work provides testable hypothesis for uncovering the functional differences between TsaC and Sua5 proteins and, more generally, provides insights into the evolutionary mechanisms driving the emergence of isofunctional enzymes.

## Materials and methods

### Extraction of Sua5 and TsaC sequences and distribution analysis

Sua5 and TsaC sequences were extracted from UniProt (version 06/2021 or version 11/2022) and UniRef (version 11/2022). TsaC/Sua5 sequences in DPANN archaea were retrieved using BLASTp searches with Pa-Sua5 protein sequence as query against all genome assemblies available (nr database, version 11/2022). The hits were filtered for the presence of KxR/SxN tetrade and one sequence per species was chosen to reduce redundancy. Following this pipeline, 5,889 bacterial, 761 archaeal and 1,318 eukaryotic sequences were recovered. The identified Sua5 or TsaC orthologs were mapped on the RNA polymerase-based universal phylogenetic tree (Da Cunha et al., 2017) or on the previously established phylogeny of DPANN archaea (Moody et al., 2022). The organisms were ranked according to the NCBI taxonomy database (as of 11/2022) or, for DPANN organisms, based on (Moody et al., 2022). The accession numbers for all sequences are available at figshare repository (doi: 10.6084/m9.figshare.22283929).

### Phylogenetic analysis

For computing the universal phylogenetic tree we chose a set of species that has been previously vetted for its ability to produce a robust phylogenetic signal using universal proteins (Da Cunha et al., 2017). The corresponding Sua5/TsaC sequences (Supplementary Tables 2, 3) were extracted from Uniprot database, aligned using MAFFT v7 with auto settings (Yamada et al., 2016). The alignment was trimmed using BMGE (Criscuolo and Gribaldo, 2010) with BLOSUM30 matrix leaving 153 positions for tree construction, corresponding only to the catalytic domain residues (the SUA5 domain was removed by the trimming software). In addition, we generated an alignment of full length Sua5 sequences that contained 470 positions. The maximum likelihood (ML) trees were inferred using IQ-TREE v1.4.3 (Trifinopoulos et al., 2016) with the TESTNEW option for model selection. The branch support was obtained using nonparametric bootstrap (100 replicates), SH-aLRT test and ultrafast bootstrap approximation (1,000 replicates; Minh et al., 2013; Kalyaanamoorthy et al., 2017) or booster (Lemoine et al., 2018).

Archaeoglobi Sua5 sequences were extracted from Uniprot (doi: 10.6084/m9.figshare.22283929) and the Maximum Likelihood tree was inferred using Phylogeny.fr web page (Dereeper et al., 2008).

### Horizontal gene transfer analysis

To screen for putative horizontally transferred genes in Archaea we extracted all sequences annotated as TsaC/Sua5 from Uniprot database (9,553 sequences, database release 2019_06). We filtered this dataset to remove sequences containing less than 100 amino acids or more than 400 amino acids as well as those without the KRSN tetrade. The remaining set of 1,275 archaeal TsaC/Sua5 sequences were classified according to their taxonomic affiliation to 13 different archaeal taxa (doi: 10.6084/m9.figshare.22283929). This allowed to identify candidate proteins in “mixed” groups containing both Sua5 and TsaC users. To reduce the impact of convergent evolution on small proteins, we focused our analyses on transfer of *sua5* genes. We used BLASTp searches against the nr protein database to identify the most similar sequences to the candidate query protein. If the best-hit species was only distantly related to the query species, the query protein was selected as candidate for HGT. The HGT candidate were subjected to phylogenetic analysis to determine their origin. The phylogenetic inferences were done as indicated above (phylogenetic analysis section).

### Protein sequence alignment

To identify Sua5-or TsaC-specific residues we aligned four representative sequences for TsaC and for TsaC-like domain from each domain of life (doi: 10.6084/m9.figshare.22283929) using MAFFT v7 with auto settings (Yamada et al., 2016).

To examine the distribution of the signature residue Pro^143^/Thr^138^ we retrieved 26,036 TsaC and Sua5 sequences from the Complete Genome Data Bank of the NCBI (database release 2015_05) using BLASTN, with Sua5 from *Pyrococcus abyssi* or TsaC from *Escherichia coli* as query. Using hmmsearch (Finn et al., 2011) we further retrieved 1,088 eukaryotic TsaC and Sua5 sequences from the Uniprot-Proteomes database (database release 2018_11). The hit sequences missing the KRSN catalytic tetrad or partial sequences were eliminated from the analysis. One UniRef 90 sequence per genus was kept for sequence alignment, 654 for prokaryotes and 564 for eukaryotes in total (accession numbers were deposited to doi: 10.6084/m9.figshare.22283929). Sequence alignment depiction was done using ESPript3 with default parameters (Robert and Gouet, 2014).

### Structure prediction and analysis

AlphaFold2 (Jumper et al., 2021) batch colab^1^ was used to predict *de novo* structures of atypical Sua5 proteins from *Archaeoglobus* archaea: *Archaeoglobus profundus* (*Ap*-Sua5, UniProt D2RFV3), *A. veneficus* (*Av*-Sua5, UniProt F2KMZ1) and *A. fulgidus* (*Af*-Sua5, UniProt O29477). Crystal structures of several TsaC and Sua5 proteins were used for structural comparison. The PDB accession codes of those structures are 1HRU and 2MX1 for *Ec*-TsaC, 2EQA, 3AJE and 4E1B for *St*-Sua5, 6F87 for *Pa*-Sua5, 3VEZ for *St*-TobZ and 3TTC for *Ec-*HypF. Structures were visualized and edited with UCSF ChimeraX v. 1.4 (Pettersen et al., 2021).

## Results

### The distribution of Sua5 and TsaC proteins does not follow a clear phyletic pattern

Previous work established that TsaC/Sua5 family of proteins (COG0009) is ubiquitous however the specific distribution of TsaC or Sua5 orthologs was only examined for a small number of representative isolated species.

**Figure 2:**
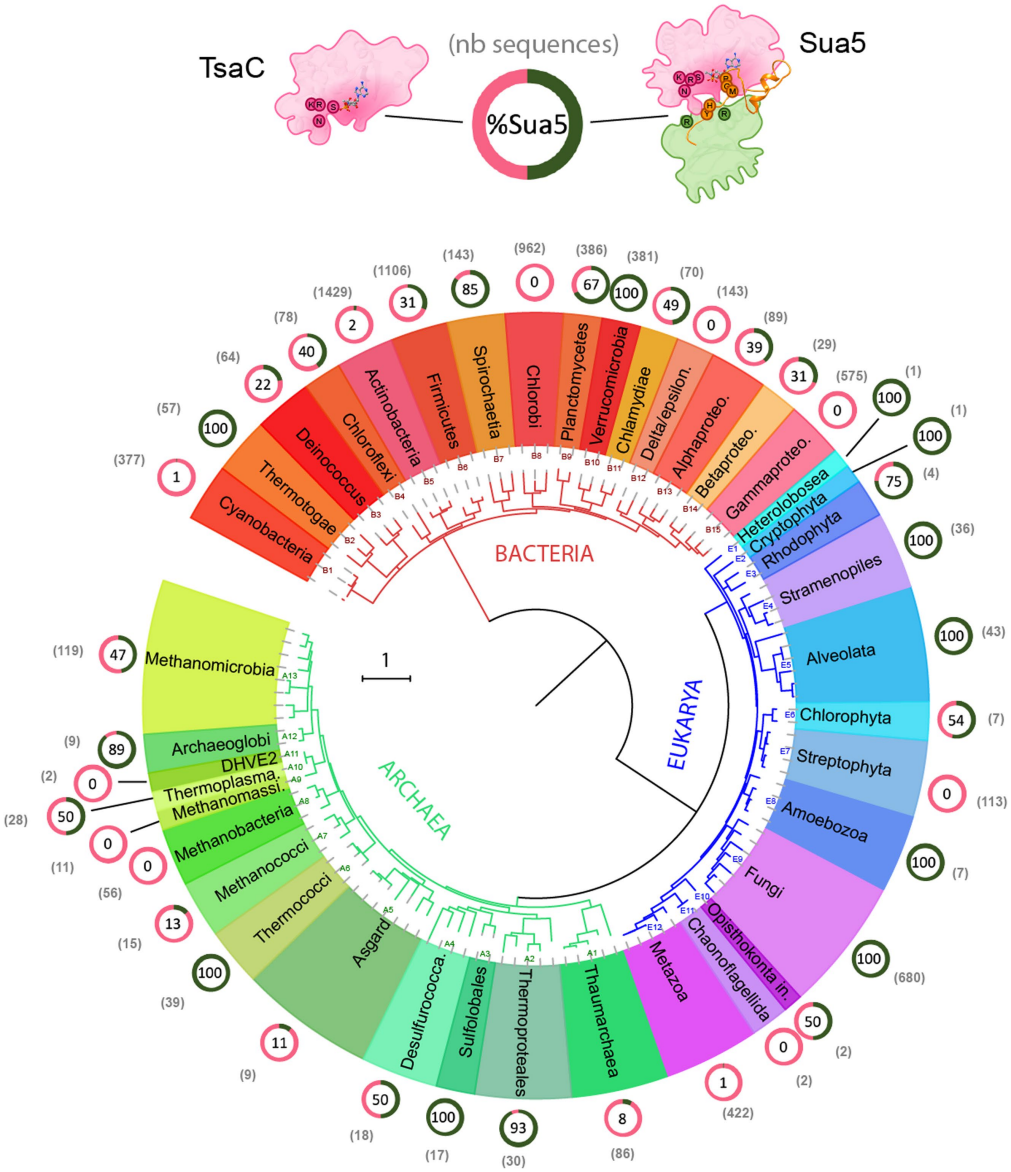
Distribution of TsaC and Sua5 proteins across the tree of life. The universal phylogeny based on RNA polymerase sequences is depicted. The number of TsaC and Sua5 sequences is indicated in the brackets for each taxon. The ring graph indicates the ratio of TsaC (pink) and Sua5 (green) orthologs for a given taxon. The numbers in the ring correspond to the percentage of Sua5 sequences. The scale bar corresponds to the number of substitutions per position in the alignment.

Here, we exploited the recent massive increase of (meta)genome sequencing data to perform an up to date comprehensive analysis of the distribution of Sua5 and TsaC proteins among living organisms. To this end, we retrieved 5,889 bacterial, 761 archaeal and 1,318 eukaryotic sequences from public databases and mapped their presence on reference phylogenetic trees (Figure 2; Supplementary Table 1; Supplementary Figure 1).

The data confirm the ubiquitous distribution of the Sua5/TsaC family among organisms. We found that the obligate ectosymbiotic archaea *Nanoarchaeum equitans*, and *Nanobdella aerobiophila*, were the only cultured organisms that lack the genes encoding TsaC/Sua5 proteins. In addition, we failed to detect Sua5/TsaC proteins in Undinarchaeota and Huberarchaea lineages from DPANN archaeal superphylum (Supplementary Figure 1) but it is unclear whether they are genuinely missing or were missed during metagenomic assemblies. Notably, in the entire tree we detected few species encoding both TsaC and Sua5: in the *Leotiomyceta* fungi clade (e.g., *Aspergillus bombycis*–Uniprot ID A0A1F7ZM40 and A0A1F8A8Q8) and in the *Vibrio* bacteria (e.g., *Vibrio aquaticus* – Uniprot A0A3S0QCR7 and A0A3S0V185). The data further show that out of 41 examined taxonomic groups depicted in Figure 2; Supplementary Figure 1, 10 groups contain species that are exclusively Sua5 users, 8 groups contain species that encode the TsaC orthologs only, 3 groups contain at least 98% of species that encode only the TsaC orthologs and the remaining 20 taxons contain species encoding Sua5 or TsaC. Intriguingly, however, the distribution of TsaC and Sua5 users across the tree shows no clear phyletic pattern suggesting a complex evolutionary history for the Sua5/TsaC family of proteins.

### The phylogeny suggests a pre-LUCA origin of TsaC/Sua5 family

**Figure 3:**
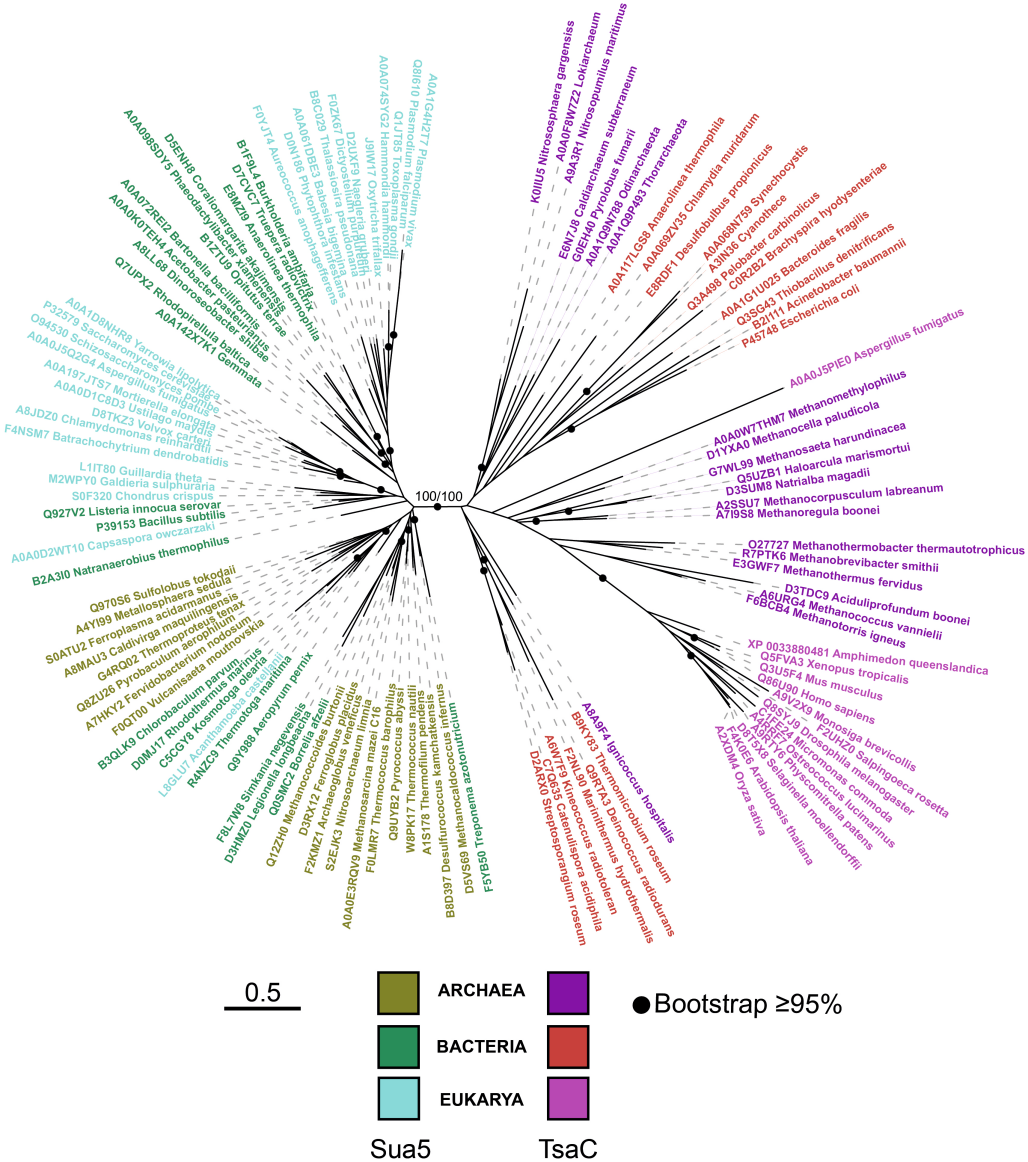
The phylogeny of the TsaC/Sua5 family of proteins. The maximum likelihood tree was generated from the alignment of representative TsaC and Sua5 sequences (see Supplementary Tables 2,3) using LG + R6 sequence evolution model. Sequences of TsaC and TsaC-like domain of Sua5 proteins were used for the alignment. The scale bar corresponds to the number of substitutions per amino acid in the alignment.

To gain insight into the evolutionary history of Sua5/TsaC family we constructed a phylogenetic tree using balanced taxonomic sampling across the three domains of life (Supplementary Tables 2, 3). The tree was inferred from the alignment of TsaC proteins and the catalytic TsaC-like domain of Sua5 proteins. It exhibits bipartite topology whereby TsaC proteins and TsaC-like domains robustly (bootstrap values 100%) segregated into two monophyletic clades (Figure 3), each clade containing sequences from the three domains of life. The branches in the TsaC part of the tree are in general longer suggesting that these proteins evolved at a higher rate. While in the Sua5 part of the tree no clear phyletic pattern can be observed, the TsaC tree shows that eukaryotic sequences cluster together and seem to be more related to archaeal TsaC sequences thus recapitulating the established phylogeny of organisms. Similarly, the tree built from the alignment of full-length Sua5 sequences (Supplementary Figure 2) revealed a clear phyletic pattern whereby the bacterial and archaeal sequences formed two distinct groups while the eukaryotic sequences were split into two clades. The bipartite tree topology suggests that Sua5 and TsaC emerged from an ancient duplication event that occurred before LUCA, followed by either SUA5 domain acquisition or loss to yield the two variants.

### Horizontal transfer of sua5 and tsaC genes occurs across a wide range of phylogenetic distances

The patchy distribution of Sua5 and TsaC proteins could, at least in part, be explained by horizontal gene transfers (HGT) of *sua5* and *tsaC* genes. To identify such events, we performed an initial screen using BLASTp searches whereby we focused on “mixed” taxonomic groups containing Sua5 users and TsaC users. For example, within Chlorophyta phylum (eukaryotes, green algae, E6 in Figure 2), the Chlorophyceae family (e.g., *Volvox carteri f. nagariensis –* Uniprot D8TKZ3) is the only one carrying Sua5 orthologs (Supplementary Table 1). This protein is most similar to the Sua5 from *Apophysomyces ossiformis* (Uniprot A0A162WET7, 57% sequence identity) which is a Mucoromycota fungus. Similarly, Rhodophyta (E3), are Sua5 users except for one species (*Rhodosorus marinus* – Uniprot A0A7S0G1B8) that encodes TsaC. This protein is most similar (49% identity) to the TsaC from Firmicutes bacteria (*Brochothrix campestris*). Leotiomyceta fungi such as *Aspergillus* are a peculiar case because several encode both Sua5 and TsaC proteins with a complete KxR/SxN motif suggesting that both orthologs are functional. However, the latter protein shows highest sequence similarity with Actinobacteria (*Amycolatopsis bartoniae,* 47.5% identity) and Proteobacteria (*Verticiella sediminum*, 41.9% identity) suggesting that this may be a case of inter-domain HGT. This initial screen identified several other candidate proteins in Bacteria and Archaea. Among those, we observed that all *Methanothermococcus* species encoded TsaC ortholog except *M. thermolithotrophicus* which carries a Sua5 ortholog (WP_018153339.1). The sequence of this ortholog is identical to Sua5 from *Methanococcus maripaludis* KA1 and the gene is encoded on a ~ 14 kbp fragment that is 99% identical to *M. maripaludis* KA1 genome (Supplementary Figure 3). This suggests a recent acquisition of the whole fragment by horizontal gene transfer.

**Figure 4:**
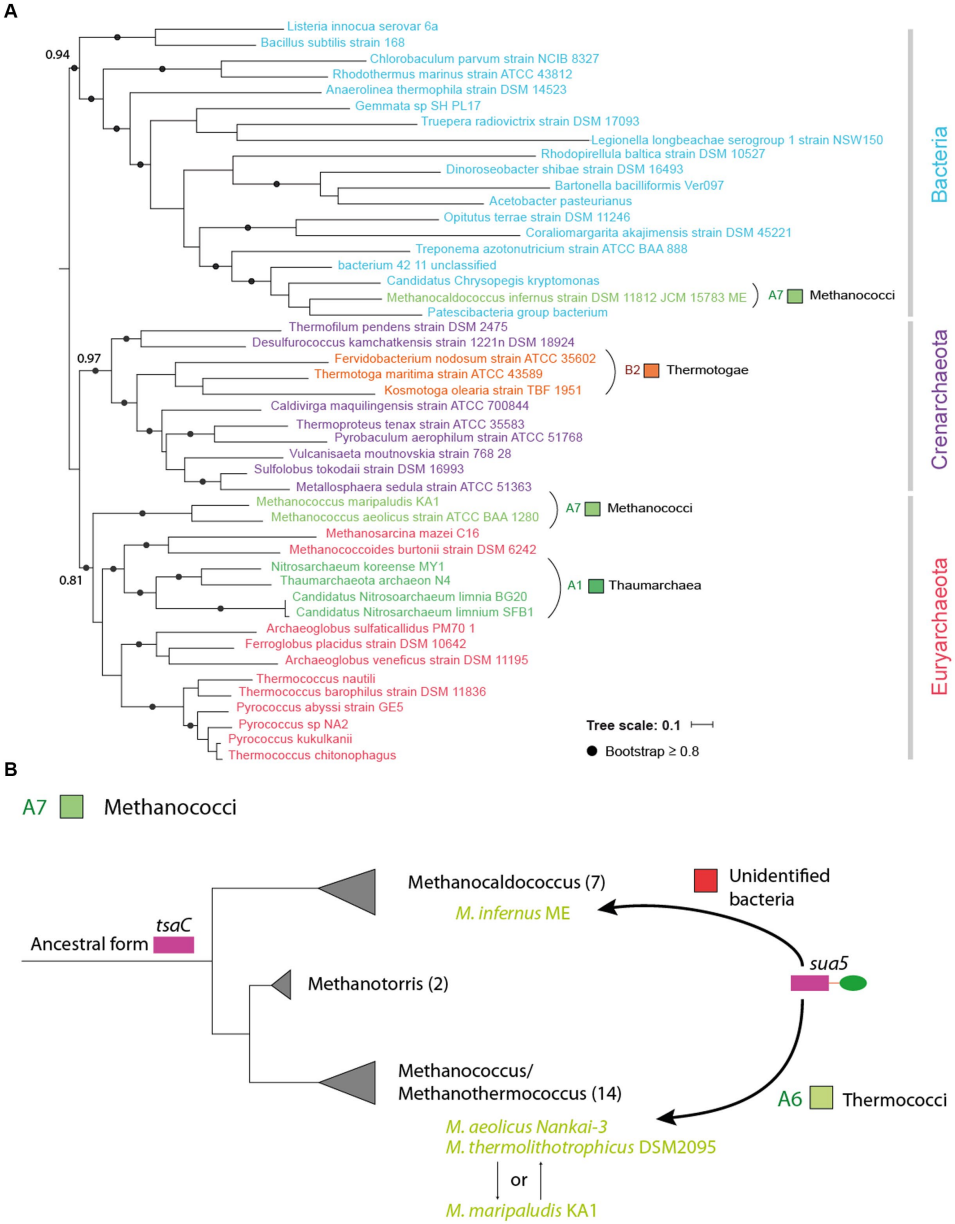
Horizontal transfer of sua5 genes occurs across different phylogenetic distances. **(A)** Maximum likelihood phylogenetic tree of Sua5 sequences from a wide range of bacterial taxa and from representative sequences of Crenarchaeota and Euryarchaeaota. The tree was arbitrarily rooted between Bacteria and Archaea. The Sua5 sequences from Methanococci, Thaumarchaea and Thermotogae are indicated in colors corresponding to their taxonomic group (see Supplementary Table 1). The scale bar corresponds to the number of substitutions per amino acid. **(B)** Evolutionary scenario for the acquisition of sua5 genes in Methanococci archaea. The established phylogeny of the four main genera of Methanococci is shown. The number of species in each genus is indicated in brackets. All species carry TsaC orthologs except for the four species (indicated in green color) that carry Sua5 orthologs. This suggest that the common ancestor of Methanococci was a TsaC-user and that the four species acquired the sua5 gene by HGT. The putative donor of these sua5 genes is indicated above the arrows.

We next tested the robustness of the BLASTp results using phylogenetic analysis. As an example, we selected outlier Sua5 proteins from groups which are predominantly TsaC-users (the Methanococci archaea (A7), Thaumarchaea (A1) and Thermotogae bacteria (B2); Supplementary Table 1) and aligned those with representative Sua5 sequences from Bacteria and Archaea. The tree was resolved into two distinct clades containing bacterial or archaeal sequences allowing to identify the closest homologs for the outlier Sua5 proteins (Figure 4A). The tree topology showed that (i) the Sua5 proteins from *Methanococcus* species and Thaumarchaeota branched within Euryarchaeota; (ii) the Sua5 protein from *Methanocaldococcus* archaeon branched within bacterial Sua5 orthologs; (iii) Sua5 proteins from Thermotoga bacteria branched within Crenarchaeal Sua5 orthologs. This suggested that the outlier Sua5 proteins were acquired by HGT from closely related (*Methanococcus*) or distantly related (Thaumarchaeota, *Methanocaldococcus*, and Thermotoga) organisms (Figure 4B).

Together, these analyses show that TsaC and Sua5 encoding genes have been submitted to horizontal transfers across different phylogenetic distances including at the highest taxonomic level.

### The identification of variant-specific and conserved residues indicates distinct modes of substrate recognition in TsaC and Sua5 proteins

**Figure 5:**
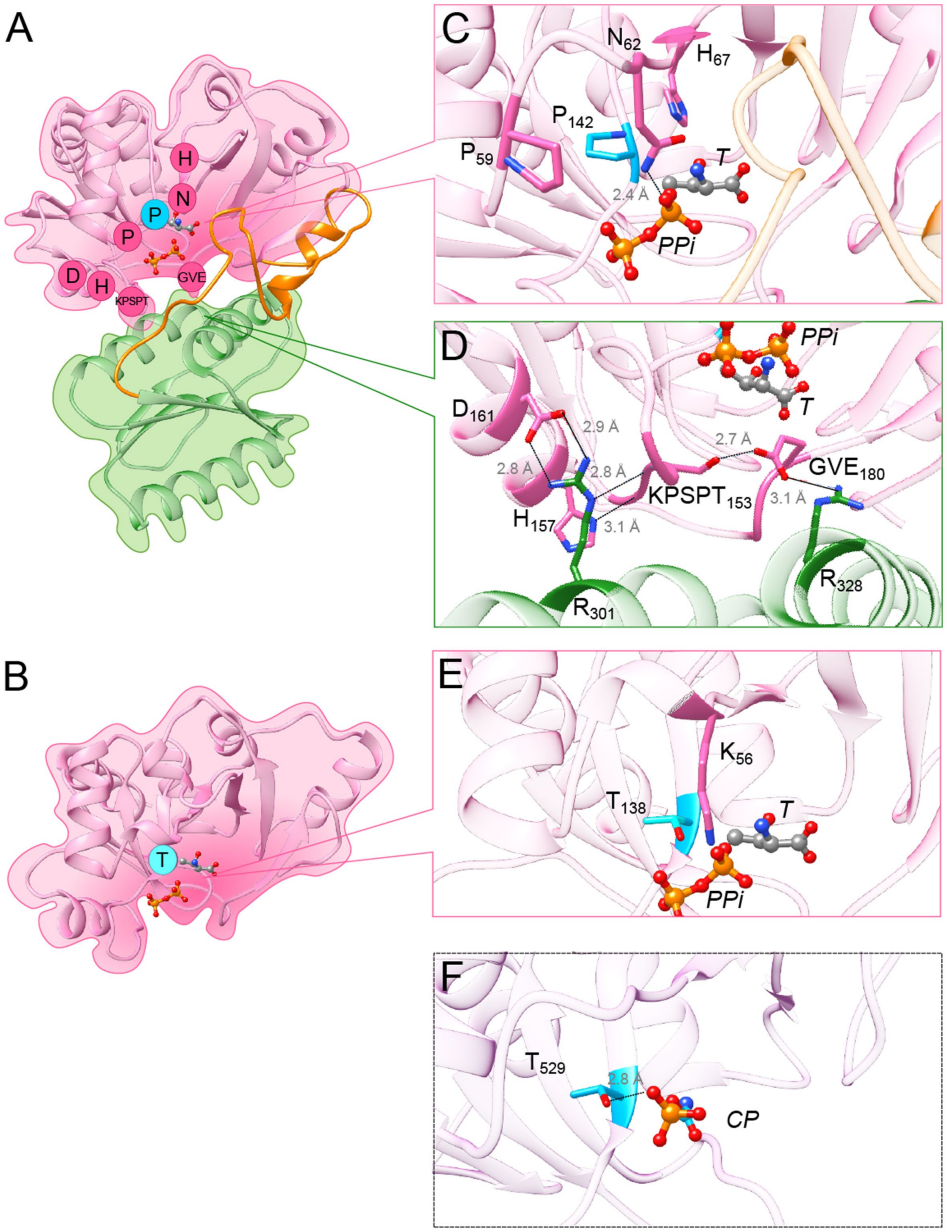
Variant-specific conserved residues interact with substrates and stabilize interdomain interface. **(A)** Cartoon showing the structure of Pa-Sua5 in complex with threonine and PPi shown as stick presentation. The TsaC-like domain is in pink, SUA5 domain is in green and the flexible loop is in orange. The Sua5-specific conserved residues are indicated with one letter code in pink circles or in cyan circle for the signature residue. **(B)** Cartoon showing the crystal structure of Ec-TsaC. Threonine and PPi molecules were modelled in the active site by superposing the Ec-TsaC with Pa-Sua5. The signature residue Thr is indicated with one code letter in a cyan-colored circle. **(C)** Zoom in the active site of Pa-Sua5. Sua5-specific conserved residues Pro^59^, Asn^62^, His^67^, and Pro^142^ are highlighted. Asn^62^ forms a hydrogen bond (indicated as dotted line) with the PPi molecule. **(D)** Zoom at the interface between the TsaC-like and SUA5 domains. The KPSPT motif and the GVE motif are shown as well as the network of H-bonds formed between these residues. Two salt bridges formed by Asp^161^ and Glu^180^ with Arg^301^ and Arg^328^, respectively, likely stabilize the two domains. **(E)** Zoom in the active site cavity of Ec-TsaC. Threonine and PPi molecules were modelled in the active site by superposing the Ec-TsaC and Pa-Sua5 that co-crystallized with these ligands. Side chain of the signature residue Thr^138^ is shown as stick model. **(F)** Zoom in the active site of TsaC-like domain of TobZ protein from Streptoalloteichues tenebrarius bound to its substrate carbamoyl-phosphate (CP). The side chain of Thr^529^ (the equivalent of Thr^138^ in Ec-TsaC) is indicated as stick model. The H-bond between Thr^529^ and the phosphate moiety is indicated by a dotted line.

The segregation of TsaC and TsaC-like sequences into two distinct phylogenetic clades indicated that each variant must contain a specific set of residues in addition to the residues conserved across the whole TsaC/Sua5 family. In search for such residues, we aligned TsaC or Sua5 sequences representative for each domain of life (Supplementary Figure 4). This showed that almost half out of approximately 200 positions in the alignment are similar, in line with a strong conservation of structure and function of the TsaC domain across the tree of life. In addition, we identified 15 conserved residues specific for Sua5 proteins (Supplementary Figure 4), 14 of which are variable among TsaC proteins. Notably, three of them, Pro^59^, Asn^62^ and His^67^ (*Pa*-Sua5 numbering) are found in the loop that forms the deep part of the catalytic cavity surface, with their side chains pointing towards the cavity (Figures 5A,C). His^67^ side chain forms a H-bond (2.7 Å) with the hydroxyl function of L-threonine in the structures of *St-*Sua5 (Supplementary Figure 5A). In the structure of *Pa*-Sua5 the side chain nitrogen of Asn^62^ acts as a donor to form a H-bond (2.4 Å) with PPi (Figure 5C) while its side chain oxygen acts as an acceptor to form a H-bond with the 3’-OH function of the ribosyl moiety of TC-AMP in the structure of *St*-Sua5 (2.6 Å; Supplementary Figure 5A). In several TsaC variants, Asn^62^ is replaced by a lysine (Supplementary Figure 4). However, unlike asparagine, lysine can only act as a H-bond donor suggesting their respective role in TC-AMP synthesis may not be identical.

Most of the other Sua5 specific conserved residues form two motifs at the inter-domain interface: the KPSPT motif is composed of (Lys/Arg)^149^-Pro^150^-Ser^151^-Pro^152^-Thr^153^ and the GVE motif is composed of Gly^178^-Hyd^179^-Glu^180^ (where Hyd corresponds to the hydrophobic residue Val, Ile or Leu; Figure 5D). The motifs interact with one another via a H-bond (2.7 Å) formed by the side chains of Ser^151^ and Glu^180^. The backbone chain of the KPSPT motif forms several H-bonds with *inter alia* the side chain of His^157^, another Sua5-specific residue, and the conserved Arg^301^ present in the SUA5 domain. In addition, the Sua5-specific amino acids Asp^161^ and Glu^180^ are forming salt bridges with Arg ^301^ and Arg^328^, respectively (Figure 5D), thus likely playing a key role in the stabilization of the inter-domain interaction.

Remarkably, only one TsaC-specific conserved residue, Thr^138^ (*Ec-*TsaC numbering), could be clearly identified (Supplementary Figure 4). This residue is found in the catalytic cavity of TsaC proteins and could potentially interact with substrate molecules (Figure 5B). To investigate this further we analyzed the structures of TsaC-like domains of TobZ and HypF proteins that co-crystalized with substrate molecules. The corresponding residue Thr^529^ in TsaC-like domain of TobZ protein forms a H-bond (2.8 Å) with the phosphor-moiety of the carbamoyl-phosphate a precursor for the formation of carbamoyl-adenylate (Figure 5F). This phosphor-moiety occupies the same position as the phosphor-moiety of TC-AMP and as the β phosphate of AMPPNP in the structures of *St-*Sua5 (Supplementary Figure 5B). In the TsaC-like domain of HypF from *E. coli*, the equivalent residue Thr^321^ interacts via a H-bond (2.6 Å) with the β phosphates of the bound ADP (Supplementary Figure 5C).

Overall, the comparative sequence and structural analyses identified variant-specific conserved residues with putative key roles in substrate binding thus highlighting previously unrecognized functional differences between TsaC and Sua5 proteins.

### A single conserved residue constitutes a distinguishing feature between Sua5 and TsaC proteins

**Figure 6:**
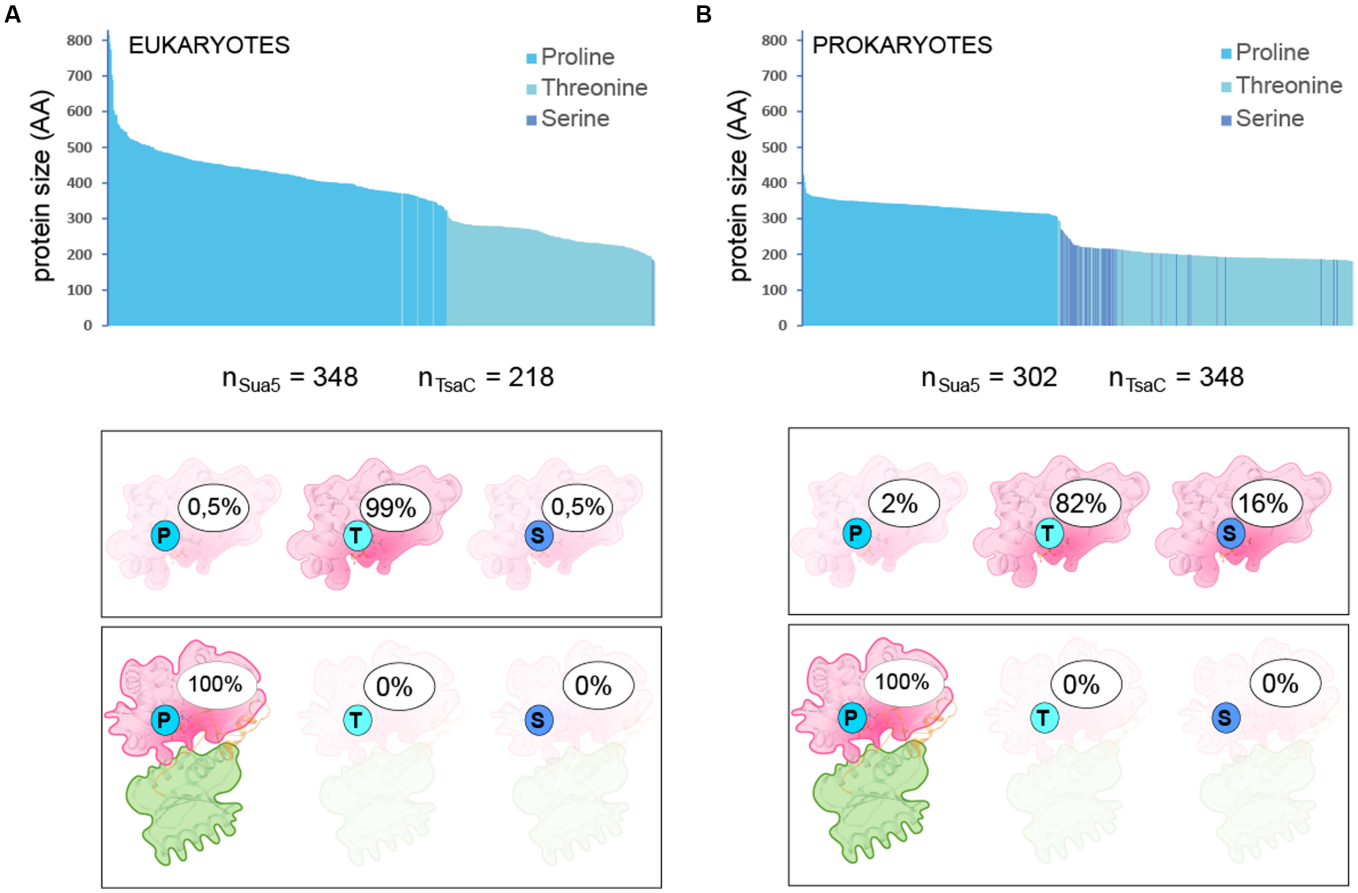
Nature and frequency of the signature residue in TsaC and Sua5 sequences Nature of the signature residue Pro^143^/Thr^138^/Ser^138^ in TsaC and Sua5 sequences from Eukaryotes **(A)** and Prokaryotes **(B)**. The top graph shows the protein length distribution whereby each sequence is represented as a vertical bar. The number of analyzed sequences is indicated under the graph. The legend gives the correspondence between the color of the bars and the presence of one of the signature residues. Bottom cartoons depict the percentage of TsaC and Sua5 having either Pro^143^, Thr^138^, or Ser^138^as the signature residue.

Structural and sequence analyses identified Thr^138^ (*Ec-*TsaC numbering) as the only conserved residues specific for TsaC proteins. Intriguingly, the corresponding residue in Sua5 proteins is a conserved Proline (Pro^143^ in *Pa*-Sua5; Supplementary Figure 4; Figure 5C) suggesting that this single and evolutionary conserved residue is a key determinant of specific mechanisms by which Sua5 and TsaC proteins catalyze the synthesis of TC-AMP. To test the relevance of this observation, we extracted 653 prokaryotic and 566 eukaryotic sequences from complete bacterial and archaeal genomes (one sequence per genus) and plotted the nature of the signature residue as a function of the size of the protein (Figure 6; Supplementary Figure 6). This showed that Sua5 proteins have exclusively Pro^143^ at this position. In TsaC sequences, Thr^138^ is predominant with up to 99% of occurrences in eukaryotes but is replaced by Ser^138^ in 16% of bacterial sequences. The only exception to this rule are TsaC sequences from the bacterial genera Brachyspira, Lactococcus, Erysipelothrix as well as the entire Aquificae phylum where Thr^138^ is replaced, intriguingly, by a Sua5-typical proline. These TsaC proteins lack the Sua5 specific motifs important for the interdomain interaction (Supplementary Figure 7) suggesting that these are genuine exceptions.

Together, these data confirm that the highly conserved Thr^138^/Ser^138^ and the corresponding Pro^143^ accurately identify TsaC and Sua5 proteins, respectively, in the vast majority of cases.

### Atypical Sua5 proteins of Archaeoglobi archaea carry functionally relevant deletions in the SUA5 domain

The identification of TsaC and Sua5 specific residues allowed us to spot atypical sequences such as those of Pro^143^-containing bacterial TsaC proteins. In addition to these outliers, our attention was drawn to the Sua5 sequence from the archaeon *Archaeoglobus profundus* (*Ap*-Sua5) that carried Thr^138^ instead of Pro^143^ signature residue and was significantly shorter (289 AA) than a classical Sua5 protein (average size 331 AA). Extending the analysis to six other isolated Archaeoglobi species showed that, in addition to *Ap*-Sua5, the Sua5 proteins from *A. veneficus* (*Av*-Sua5) and *A. fulgidus* (*Af*-Sua5) carry deletions in the interdomain loop and in the SUA5 domain (Supplementary Figure 8). Around 50% of the interdomain loop residues are missing in these proteins and among those the highly conserved and functionally important motif Pro^228^-Gly^229^-Met^230^. Moreover, when comparing with the archetypal Sua5 from *A. sulfaticallidus* these three proteins lack 12%, (*Av*-Sua5), 13% (*Af*-Sua5), and 36% (*Ap*-Sua5) of residues in the SUA5 domain.

**Figure 7:**
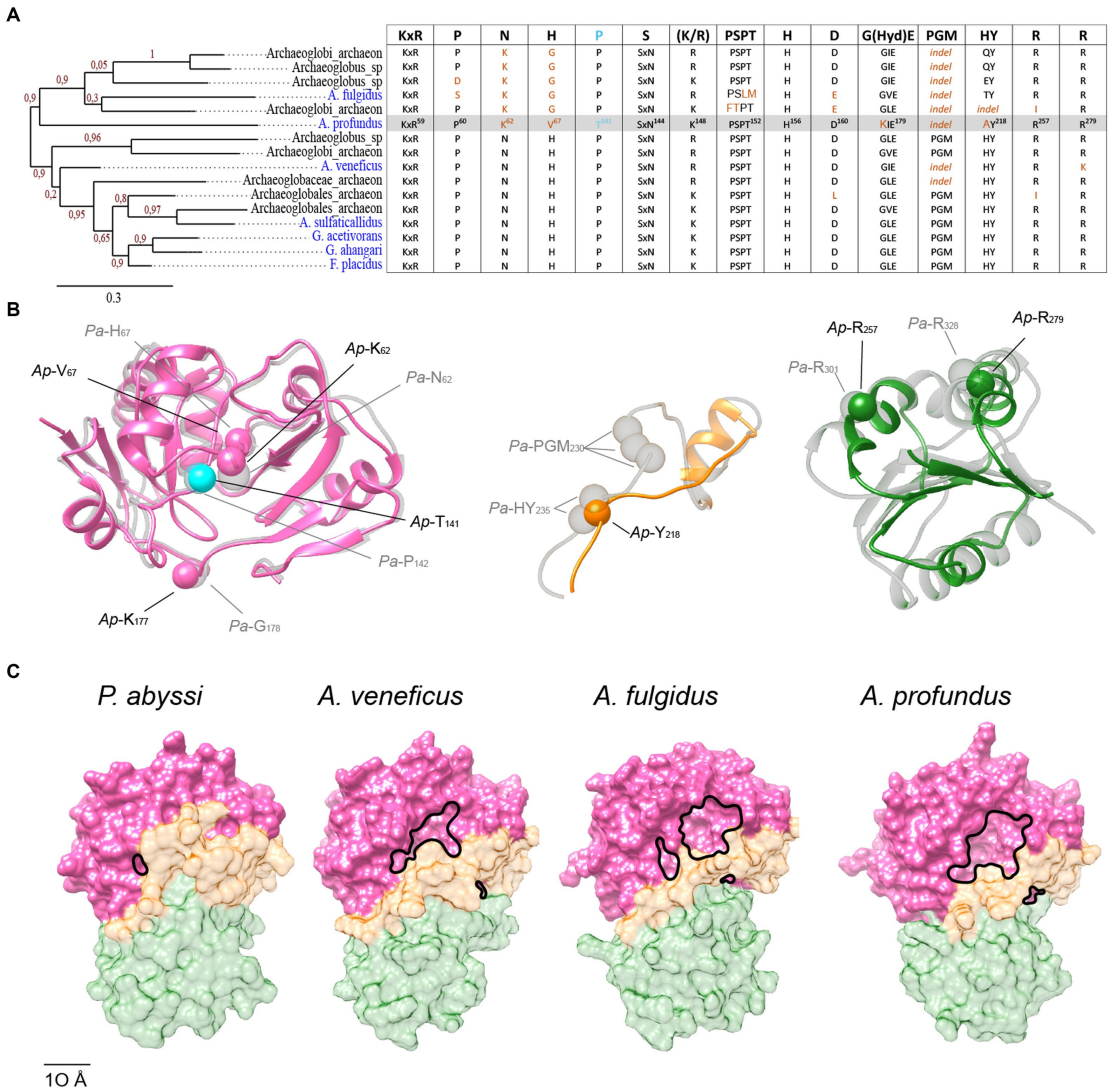
Progressive erosion of the SUA5 domain in Sua5 proteins from Archaeoglobus archaea. **(A)** Maximum likelihood tree of Sua5 proteins from Archaeoglobi species. The isolated species are highlighted in blue color. Bootstrap values for branch support are indicated. The table on the right shows the occurrence of the Sua5-specific conserved residues in the analyzed sequences. The canonical residues are indicated on the top of the table. The residues found in Ap-Sua5 are highlighted in grey and signature residue Pro^143^/Thr^138^ is indicated in cyan. Residues diverging from the consensus sequence and indels are in orange. **(B)** AlphaFold2 model of Ap-Sua5 (in color) is superposed onto the crystal structure of Pa-Sua5 shown in light gray. The consensus Sua5-specific residues and the corresponding residues found in Ap-Sua5 are indicated as spheres. **(C)** Structure of Sua5 proteins shown as molecular surface. The crystal structure of Pa-Sua5 was retrieved from the PDB database while the structures of Av-Sua5, Af-Sua5, and Ap-Sua5 were modeled using AlphaFold2. TsaC-like domain, the interdomain loop, and SUA5 domain are depicted in pink, orange, and green color, respectively.

To get insight into the history of the acquisition of the detected mutations and their potential consequences for the function of these atypical Sua5 proteins, we performed phylogenetic analyses and AlphaFold2 modelling. The sequences that accumulated the highest number of mutations clustered within a single clade (Figure 7A). Of note, they all carry a deletion in the interdomain loop leading to the loss of the PGM motif and, concomitantly, the substitution of Asn^62^ to Lys and His^67^ to Gly/Val, the residues that we have tentatively identified as being involved in interaction with substrates (Figures 5C, 7A). We next modelled the structure of *Ap*-Sua5 and compared it to the crystal structure of *Pa*-Sua5. This showed that the TsaC-like domain adopts a typical fold whereby Lys and Val residues superpose well with Asn^62^ and His^67^, respectively, suggesting that these mutations affect the binding of substrates (Figure 7B). Despite a significant shortening, the SUA5 domain of *Ap*-Sua5 was modelled as a SUA5-like globular fold with the two arginines (Arg^257^ and Arg^279^ in *Ap*-Sua5) being correctly positioned to build salt bridges with the catalytic domain (Figure 7B). However, the interdomain loop seem to adopt a much more linear conformation leaving the entrance to the catalytic cavity open to the solvent (Figure 7C).

Together, the data suggest a progressive accumulation of mutations in interdomain loop and SUA5 domain of Archaeoglobi Sua5 with *Ap*-Sua5 being the extreme case. Consequently, these atypical Sua5 proteins seem to have acquired compensatory mutations in the catalytic domain that probably affect the binding to substrates and/or TC-AMP molecules.

## Discussion

In the present work, we delineated the evolutionary pathway that led to the existence of two partially homologous proteins performing the same essential function in all extant organisms. We show that the evolution resulted in two different substrate binding modalities with potential consequences for the catalytic mechanism and/or efficiency of Sua5 and TsaC enzymes.

Early comparative genomics analyses identified TsaC/Sua5 family of proteins as part of a small set of about 60 truly ubiquitous proteins that were probably present in the Last Universal Common Ancestor (LUCA) of all extant organisms (Galperin, 2004). Our up to date distribution analysis shows that this is still true. The only fully sequenced organisms lacking *tsaC* or *sua5* genes are obligate ectosymbionts such as the DPANN archaea *N. equitans* and *N. aerobiophila*. These organisms carry greatly reduced genomes (0.49 Mbp and 0.67 Mbp, respectively) and depend on their hosts for the uptake of essential metabolites such as nucleotides, lipids and ATP (Huber et al., 2002; Waters et al., 2003; Kato et al., 2022). This suggests that the absence of *tsaC* and *sua5* genes in a genome could be a marker of symbiotic or parasitic lifestyle. It remains to be investigated whether these organisms adapted to translate their genetic information faithfully in the absence of t^6^A-modified tRNA or if this function is supplied by the host. The latter may be the case for *N. equitans* which encodes the KEOPS complex (Waters et al., 2003) suggesting that it synthetizes t^6^A-tRNA but needs the host to supply TC-AMP or the TsaC/Sua5 enzyme by some yet unknown mechanism. The transport of TC-AMP from the host *Acidianus hospitalis* to *N. equitans* seems unlikely since this molecule is highly instable at 90°C, the optimal growth temperatures of these organisms (Lauhon, 2012). The export of Sua5 enzyme from the cytoplasm to mitochondria via a signal sequence has, however, been reported, in *S. cerevisiae* (Thiaville et al., 2014a) suggesting that uptake of Sua5/TsaC by *N. equitans* could be a possible mechanism.

Our data further show that the concomitant presence of the *tsaC* and *sua5* genes in a genome is rare in accordance with previous work reporting the co-occurrence of these genes only in *Acetobacterium woodii* and *Vibrio cholerae* strains among 9,200 analyzed genomes (Thiaville et al., 2014b). In the additional cases we detected, we found that the second gene was always of exogenous origin suggesting that most of the co-occurrence cases are explained by recent horizontal gene transfers (HGT). These transfers can occur even over highest phylogenetic distances, between two different domains, suggesting that TsaC/Sua5 proteins are not dependent upon a particular cellular context and/or partners to function. This is further corroborated by functional complementation experiments showing that *tsaC* and *sua5* genes originating from very distantly related organisms (yeast, bacteria and archaea) are all interchangeable *in vitro* and *in vivo* (El Yacoubi et al., 2009; Perrochia et al., 2013a). Such “independency” would probably facilitate horizontal gene transfers of *sua5* and *tsaC* genes and this may explain, at least in part, the patchy distribution of these genes across the universal tree.

Present day distribution of the TsaC/Sua5 family is difficult to reconcile with a simple evolutionary scenario. The bipartite tree topology of this family resembles that of translation elongation factors EF-Tu and EF-G which are paralogs that duplicated before the divergence of all extant organismal lineages (Iwabe et al., 1989; Baldauf et al., 1996). Therefore, the phylogeny indicates that TsaC/Sua5 family emerged in a pre-LUCA ancestor and that at the time of LUCA both *tsaC* and *sua5* existed. However, several observations we made contradict this scenario. First, TsaC-like domain of Sua5 proteins contains 15 conserved residues (10% of the alignment positions used for tree construction) that are variable in the TsaC proteins. We therefore cannot exclude the possibility that the bipartite tree topology we observe is artificial and simply reflects the fact that TsaC and TsaC-like sequences are “mechanically” segregated. Second, we identified several clear cases of HGT of *sua5* or *tsaC* genes whereby the endogenous gene was lost suggesting that the concomitant presence of the two genes carrying out the same function is not advantageous for an organism. The presence in many bacterial lineages of an inactive paralog of TsaC called YciO (Thiaville et al., 2014b) further supports this idea. Finally, the concomitant presence of both enzymes in LUCA would imply numerous independent gene loss events throughout the tree of life in order to explain the presence of only one of the two genes in extant organisms. In the light of these observations we therefore favour a more parsimonious scenario whereby LUCA encoded one of the two enzymes.

Which of the two variants is then the version that was present in LUCA? One possibility is the emergence of *tsaC* first and *sua5* post-LUCA by acquisition of an additional SUA5 domain. Domain fusion is a frequent method for new proteins to arise (Bornberg-Bauer et al., 2010), with evolution famously being described as a tinkerer rather than an inventor (Jacob, 1977). The SUA5 domain contains an atypical Rossmann fold which is an ancient and widely distributed protein fold (Ma et al., 2008) and therefore we cannot exclude a reassignment of this domain into a SUA5 domain. Of note, the presence of highly conserved motifs at the interface between the two domains supports the idea that all current Sua5 proteins originated from one common ancestral protein suggesting that the SUA5 domain acquisition, if it occurred, was a unique event.

The emergence of *tsaC* from *sua5* could have happened through the introduction of a premature stop codon. However, such drastic event may disrupt stability and/or activity of a protein (Weiner et al., 2006) and we observed this for truncated version of *Pa-*Sua5 whereby the whole SUA5 domain was removed (Pichard-Kostuch et al., 2018). Rather, a progressive accumulation of deletions in SUA5 domain such as observed in atypical Archaeoglobi Sua5 could be a mechanism that initially led to emergence of TsaC. This process would need to be accompanied by mutations compensating for the loss of the loop and the SUA5 domain. Archaeoglobi Sua5 indeed presents non-neutral mutations of highly conserved Sua5-specific residues (Asn^62^/Lys^62^ and His^67^/Gly or Val^67^). Given their position in the active site, we speculate that these mutations affect the substrates and/or product binding.

The gene erosion to yield shorter TsaC variant may have occurred several times in the course of evolution and each time a different compensatory set of mutations could have arisen thus explaining the absence of highly conserved TsaC-specific residues. This makes the TsaC-specific and conserved Thr/Ser^138^, the sole exception to this rule, particularly interesting as it suggests that this residue is a key prerequisite for becoming the shorter version of the enzyme. The comparative structural analysis performed here suggests that Thr/Ser^138^ contacts the alpha phosphate moiety of ATP/TC-AMP. Intriguingly, the corresponding Sua5 residue Pro143 would not be able to establish such a contact. However, the highly conserved histidine in the HY motif found in the interdomain loop was shown to be important for the activity of *Pa-*Sua5, likely by being involved in the binding of PPi/ATP (Pichard-Kostuch et al., 2018). Notably, none among the thousands of Sua5/TsaC sequences we screened in our analysis displayed both of these residues. It is therefore tempting to suggest that simultaneous presence of Thr/Ser^138^ and HY motifs, both of which may interact with PPi/ATP, could have a negative impact on catalysis. Consequently, this combination was counterselected, resulting in the two variant-specific signature residues. Some evidence that this may be a plausible scenario comes from the atypical Sua5 protein of *Archaeoglobus profundus*, the most advanced case of SUA5 domain erosion, where the Pro^143^ signature residue was replaced by a threonine.

The Sua5 protein of *A. profundus* would be a good candidate to test whether a Sua5 protein could be evolved to become TsaC. Using directed mutagenesis it could be possible to generate even shorter active variants and ultimately remove the SUA5 domain completely. If our hypothesis is true than the complete loss of the SUA5 domain would only be possible in combination with further mutations of substrate binding residues to their TsaC-like counterparts. As a further line of experimental studies, it would be interesting both from the evolutionary and mechanistic standpoint, to compare the specific activities of TsaC and Sua5 proteins from closely related organisms.

In conclusion, we suggest that Sua5 was the ancestral version of the extant TC-AMP synthetic enzyme family. By articulating our previous experimental observations (Pichard-Kostuch et al., 2018) with our new in-depth sequence-structure analysis of this family, we suggest that the SUA5 domain is still essential for the activity of Sua5 variant by *inter alia* ensuring the right positioning of the catalytically important linker. However, key mutations in the TsaC-like domain can reduce this dependency and lead to the emergence of TsaC from Sua5 through SUA5 domain loss. Although it remains to be tested how these mutations affect the activity, this scenario combined with HGT events would account for the present day broad but inconsistent distribution of *tsaC* and *sua5* genes among different lineages. We suggest that SUA5 domain loss occurred at several independent occasions and that the resulting TsaC proteins adapted by evolving different interfaces for binding to substrates and/or products. Thus, TsaC proteins could be a more “advanced” version of the TC-AMP producing enzyme suggesting that, to quote Leonardo da Vinci, “simplicity is the ultimate sophistication” when it comes to the evolution of Sua5/TsaC family.

## Data availability statement

The datasets presented in this study can be found in online repositories. The names of the repository/repositories and accession number(s) can be found at: https://figshare.com/, doi: 10.6084/m9.figshare.22283929.

## Author contributions

APK, VDC and TBL conceived the study and all authors analysed the data. APK, VDC and TBL prepared the figures, wrote the draft and finalized the manuscript. All authors proofread the manuscript and approved the submitted version.

## Funding

This work was funded by Agence Nationale de la Recherche, grant number ANR-18-CE11-0018 to TB.

## Conflict of interest

The authors declare that the research was conducted in the absence of any commercial or financial relationships that could be construed as a potential conflict of interest.

## Publisher’s note

All claims expressed in this article are solely those of the authors and do not necessarily represent those of their affiliated organizations, or those of the publisher, the editors and the reviewers. Any product that may be evaluated in this article, or claim that may be made by its manufacturer, is not guaranteed or endorsed by the publisher.

## Footnotes

^1^https://colab.research.google.com/github/sokrypton/ColabFold/blob/main/batch/AlphaFold2_batch.ipynb
