# Supplementary material for: The universal Sua5/TsaC family evolved different mechanisms for the synthesis of a key tRNA modification
Source: Front Microbiol. 2023 Jun 21;14:1204045. doi: 10.3389/fmicb.2023.1204045 (PMC10321239; doi:10.3389/fmicb.2023.1204045)
Supplement: Supplementary file 1 [file Data_Sheet_1.PDF]

**Supplementary Information for**

# The universal Sua5/TsaC family evolved different mechanisms for the synthesis of a key tRNA modification

Adeline Pichard-Kostuch[1,$], Violette Da Cunha[1,£], Jacques Oberto[1], Ludovic Sauguet[2] & Tamara Basta[1,#].

[1] Université Paris-Saclay, CEA, CNRS, Institute for Integrative Biology of the Cell (I2BC), 91198, Gif-sur-Yvette, France

[2] Institut Pasteur, Unité de Dynamique Structurale des Macromolécules, 75015 Paris, France

[#]Corresponding author: tamara.basta@i2bc.paris-saclay.fr

[£]Present address: Génomique Métabolique, Genoscope, Institut François Jacob, CEA, CNRS, Univ Evry, Université Paris-Saclay, 91057 Evry, France

SUPPLEMENTARY FIGURES AND TABLES

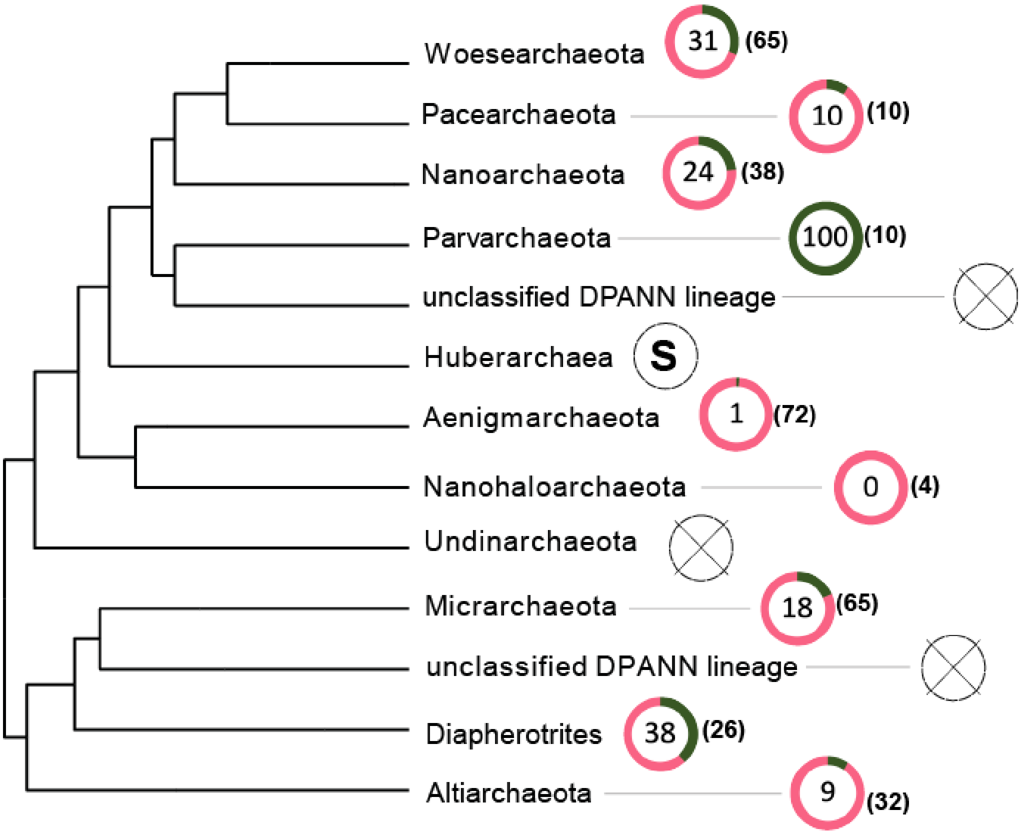


**Supplementary figure 1. Distribution of TsaC and Sua5 proteins in the DPANN archaea.**

Cladogram representing the phylogeny of DPANN archaea. The number of TsaC and Sua5 sequences is indicated in the brackets for each taxon. The ring graph indicates the ratio of TsaC (pink) and Sua5 (green) orthologs for a given taxon. The numbers in the ring correspond to the percentage of Sua5 sequences. Black dotted circle in the DPANN tree indicates that TsaC/Sua5 sequences could not be identified. A capital S indicates, the potential symbiotic nature of this taxon.

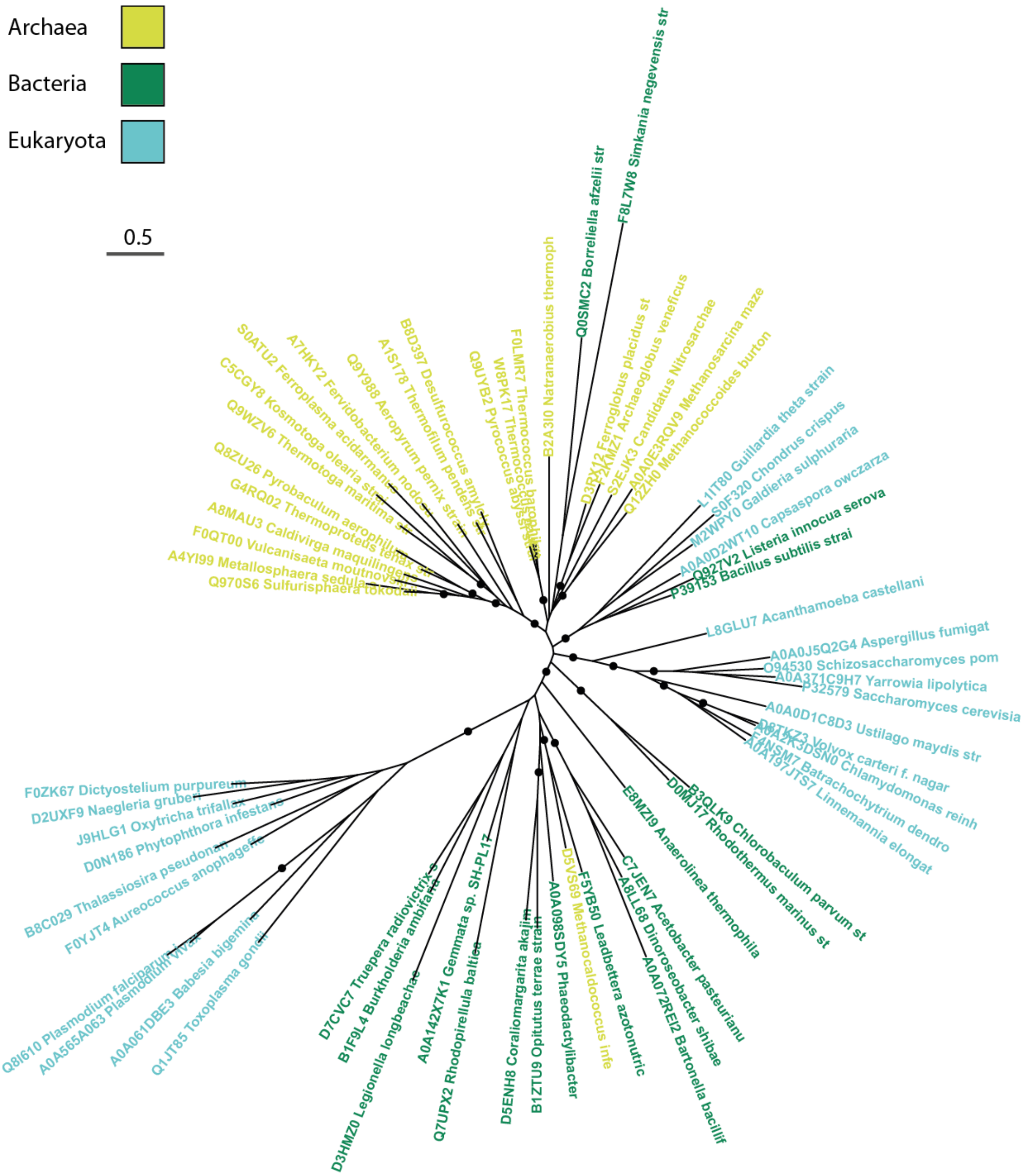


**Supplementary figure 2. The phylogeny of Sua5 proteins.**

The maximum likelihood tree was generated from the alignment of representative full-length Sua5 sequences (see Supplementary Table 2) using LG+R7sequence evolution model. The scale bar corresponds to the number of substitutions per amino acid in the alignment.

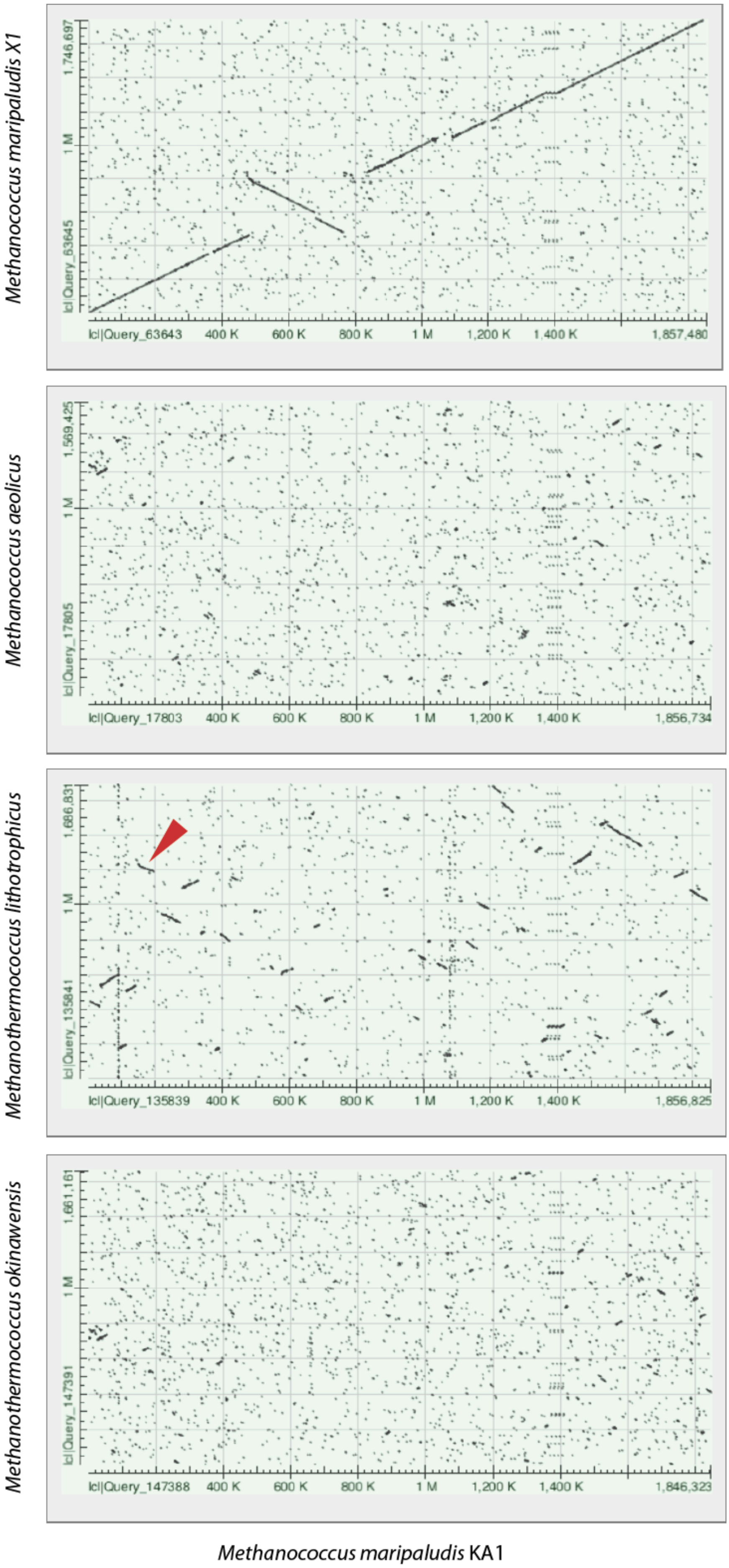
Methanococcus maripaludis X1
lcl|Query_63645
1 M
1,746,697
lcl|Query_63643
400 K
600 K
800 K
1 M
1,200 K
1,400 K
1,857,480
Methanococcus aeolicus
lcl|Query_17805
1 M
1,569,425
lcl|Query_17803
400 K
600 K
800 K
1 M
1,200 K
1,400 K
1,856,734
Methanothermococcus lithotrophicus
lcl|Query_135841
1 M
1,686,831
lcl|Query_135839
400 K
600 K
800 K
1 M
1,200 K
1,400 K
1,856,825
Methanothermococcus okinawensis
lcl|Query_147391
1 M
1,661,161
lcl|Query_147388
400 K
600 K
800 K
1 M
1,200 K
1,400 K
1,846,323
Methanococcus maripaludis KA1

**Supplementary figure 3. Whole genome pairwise sequence similarity analysis**
Whole genome pairwise BLASTn search was conducted using default parameters and the results are shown as dotblots. Black lines correspond to significantly similar genomic regions (identities > 75%, E values = 0). The axes correspond to genome size in nucleotides. The red arrowhead indicates the ~14 kbp genomic fragment encoding Sua5 protein in *M. maripaludis* KA1 and *M. lithotrophicus*. Genome accession numbers are OX296583 (*M. lithotrophicus*), CP002913.1 (*M. maripaludis* X1), CP104873.1 (*M. aeolicus*), CP002792.1 (*M. okinawensis* IH1) and AP011526.1 (*M. maripaludis* KA1).

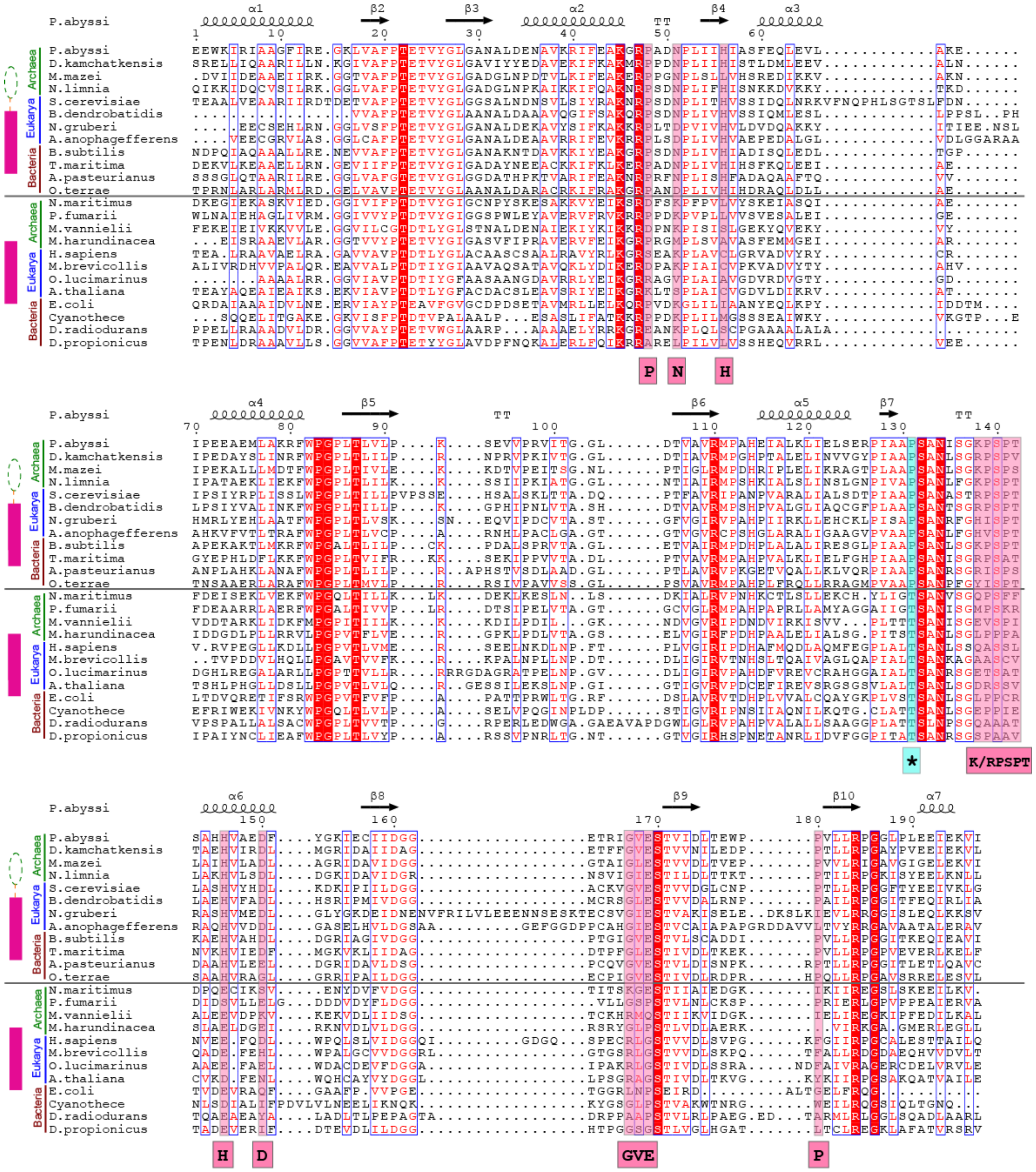

**Supplementary figure 4. Alignment of the representative Sua5 and TsaC sequences.** The sequences of the TsaC proteins and the catalytic domains of the TsaC-like domain of Sua5 proteins were aligned with MAFFT and the alignment was depicted using ESPript3. The crystal structure of Sua5 from *P. abyssi* (PDB 6F87) was used as reference to compute the distribution of secondary structures (indicated on top of the alignment). Identical positions are boxed in red, the positions with global similarity score higher that the threshold value of 0.7 are in red letters and framed in blue. The residues conserved only in TsaC-domains of Sua5 proteins are highlighted in pink and the consensus residue is indicated below the alignment. The asterisk and cyan colour indicate the signature residue Thr142/Pro142. We could not identify other conserved residues found exclusively in TsaC proteins. Note that the numbering of the alignment is off by 11 residues which were removed from the analysis for better display of the alignment.

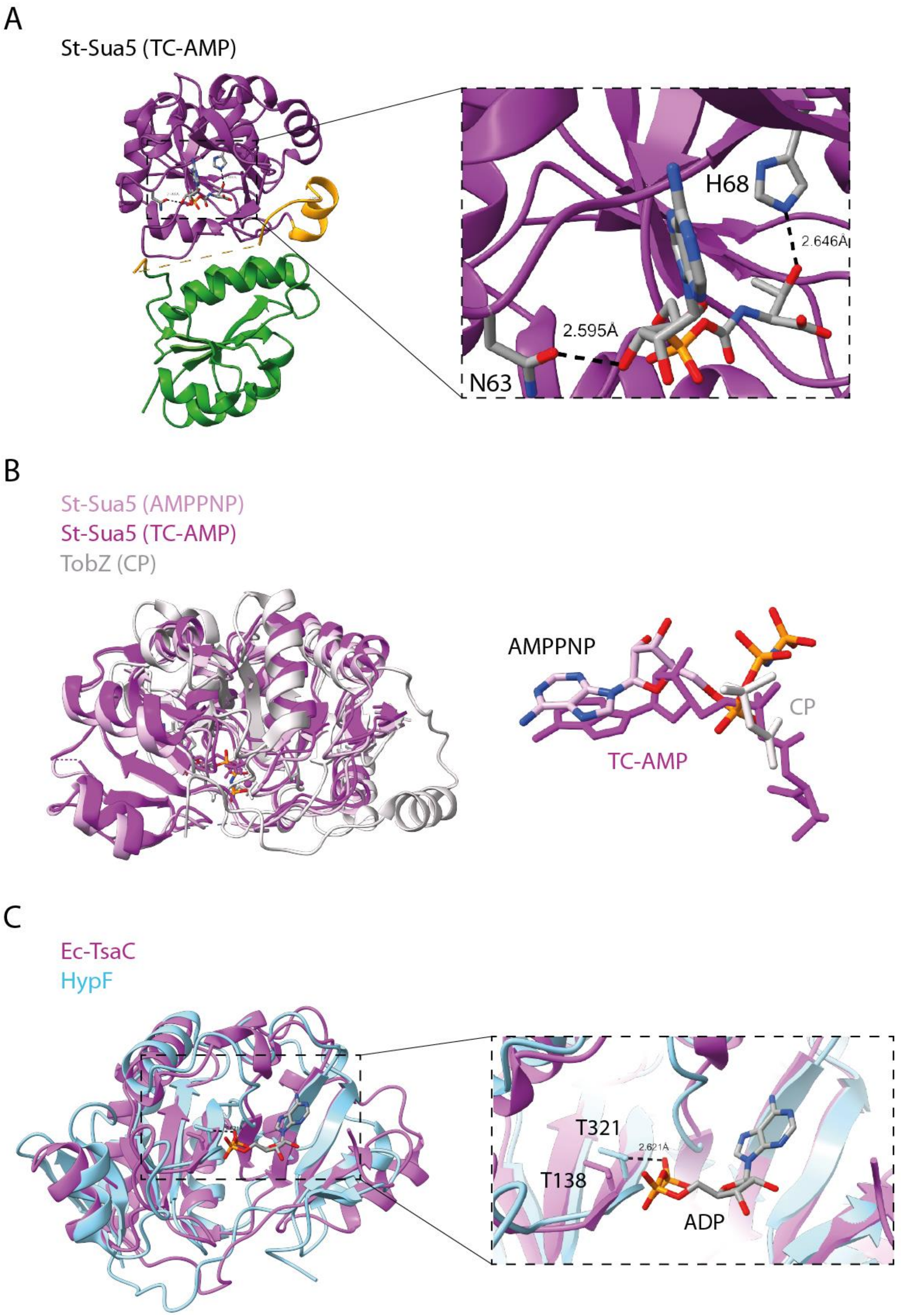


**Supplementary figure 5. Comparison of TsaC/Sua5 with HypF and TobZ structures.** A) Structure of St-Sua5 bound to TC-AMP (PDB 4E1B) is shown on the left. On the right, zoom to the catalytic cleft showing that the conserved Sua5-specific residues Asn$^{63}$ and

His$^{68}$ form hydrogen bonds (dashed line) with 3'OH of the ribosyl moiety and the side chain of the threonyl moiety of TC-AMP. B) On the left, overlay of St-Sua5 structures bound to AMPPNP (PDB 3AJE) or TC-AMP (PDB 4E1B) and TobZ structure (PDB 3VEZ). For clarity, only the TsaC-like domains are shown. On the right, only the overlay of the bound ligands is shown. CP stands for carbamoyl phosphate; TC-AMP is threonyl-carbamoyl AMP and AMPPNP is s a non-hydrolysable analogue of ATP. C) On the left, overlay of Ec-TsaC (PDB 1HRU) and the TsaC-like domain of HypF bound to ADP (PDB 3TTC). The zoom of the catalytic cavity is shown on the right. A dashed line indicates the hydrogen bond between the Thr321 and beta phosphate moiety of ADP.

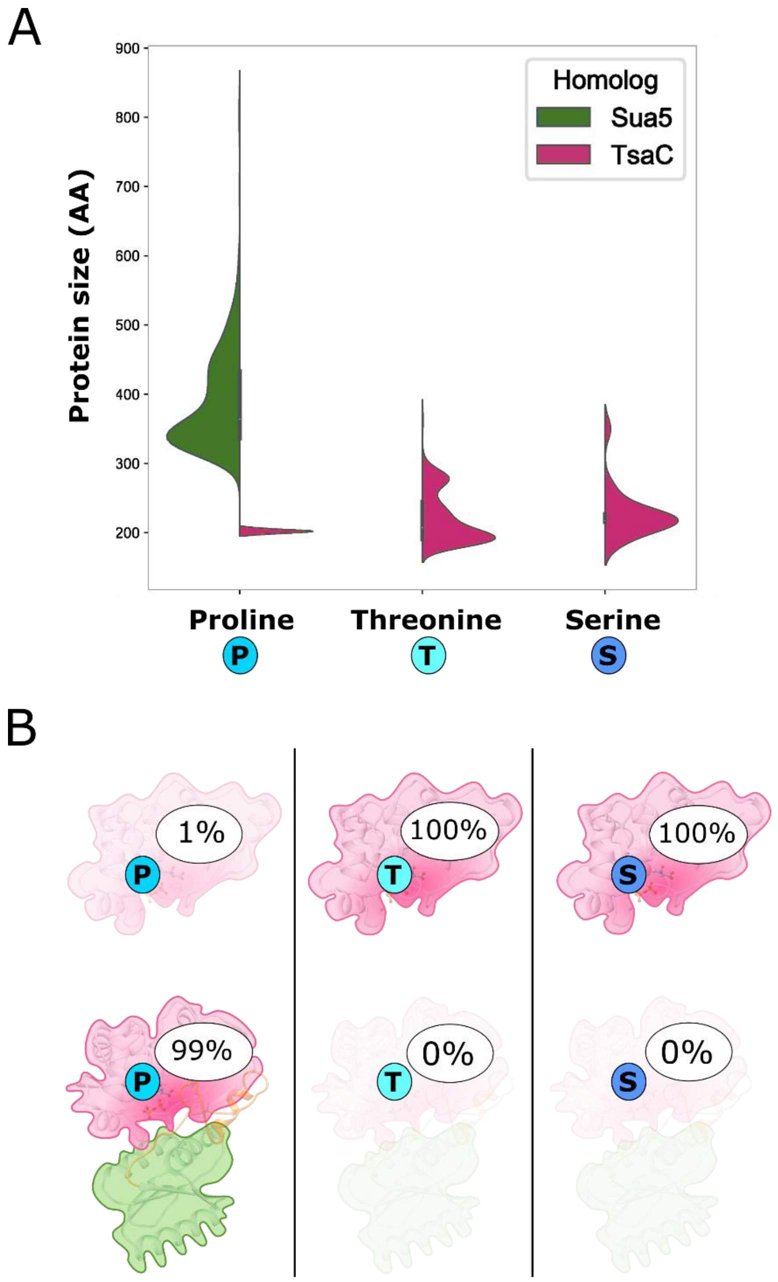


**Supplementary figure 6. Occurrence of a signature residue in TsaC and Sua5 proteins**

A) TsaC and Sua5 sequences from completely sequenced bacterial and archaeal genomes (26.036 sequences) as well as eukaryotic sequences (1088 sequences) were retrieved from the NCBI database. One sequence per genus was retained for the analysis yielding a total of 1216 sequences. The sequences were aligned using MAFFT to identify the signature residue and classified according to the nature (Pro, Thr or Ser) of the signature residue. The protein length distribution for each class of sequences was plotted as violin plot. The green and pink color correspond to Sau5 and TsaC proteins, respectively. B) Summary of the signature residue distribution in TsaC and Sua5 proteins. The occurrence of signature residues within TsaC proteins or TsaC-like domains (shown as cartoon) is indicated in percentage.

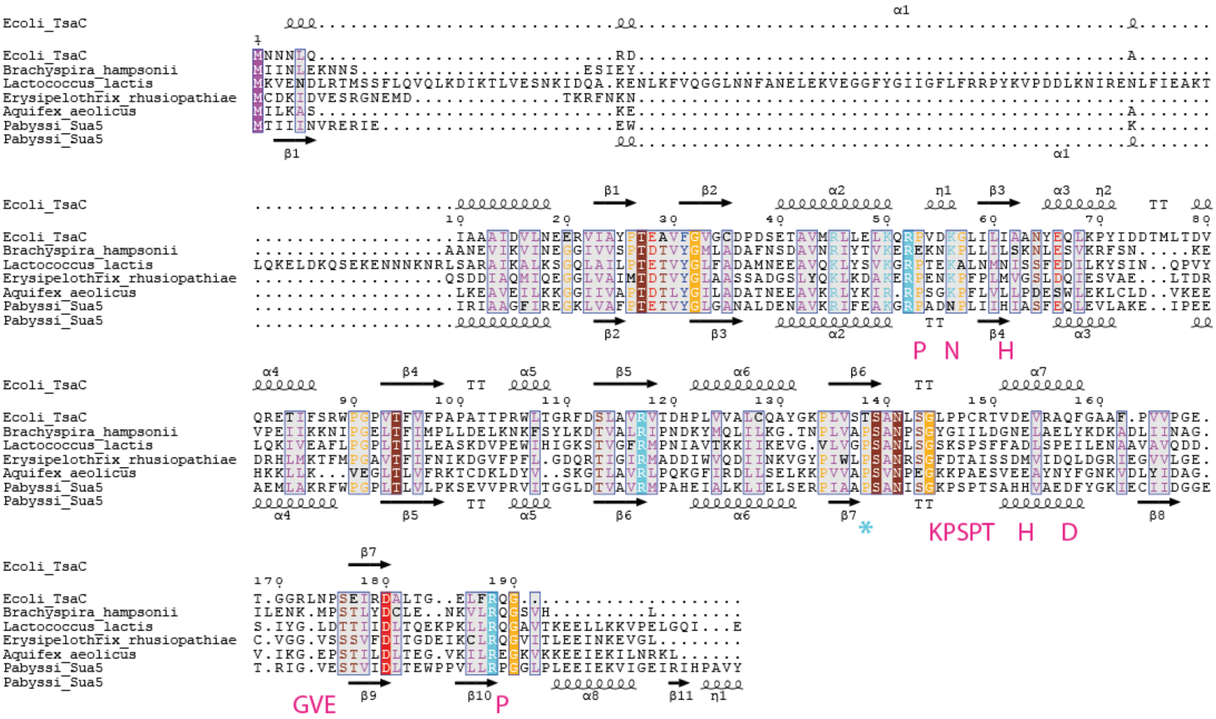

**Supplementary figure 7. Bacterial TsaC proteins with proline as a signature residue.**

The sequences were retrieved from UniProt and NCBI database, aligned with T-coffee and rendered using EPsspript. Ec-TsaC structure (PDB 1HRU) and Pa-Sua5 structure (PDB 6F87) were used as references for structural alignment. The residues were colored by similarity. The Sua5-specific conserved residues are indicated below the alignment in pink color. The signature residue Pro143 is highlighted with a cyan asterisk.

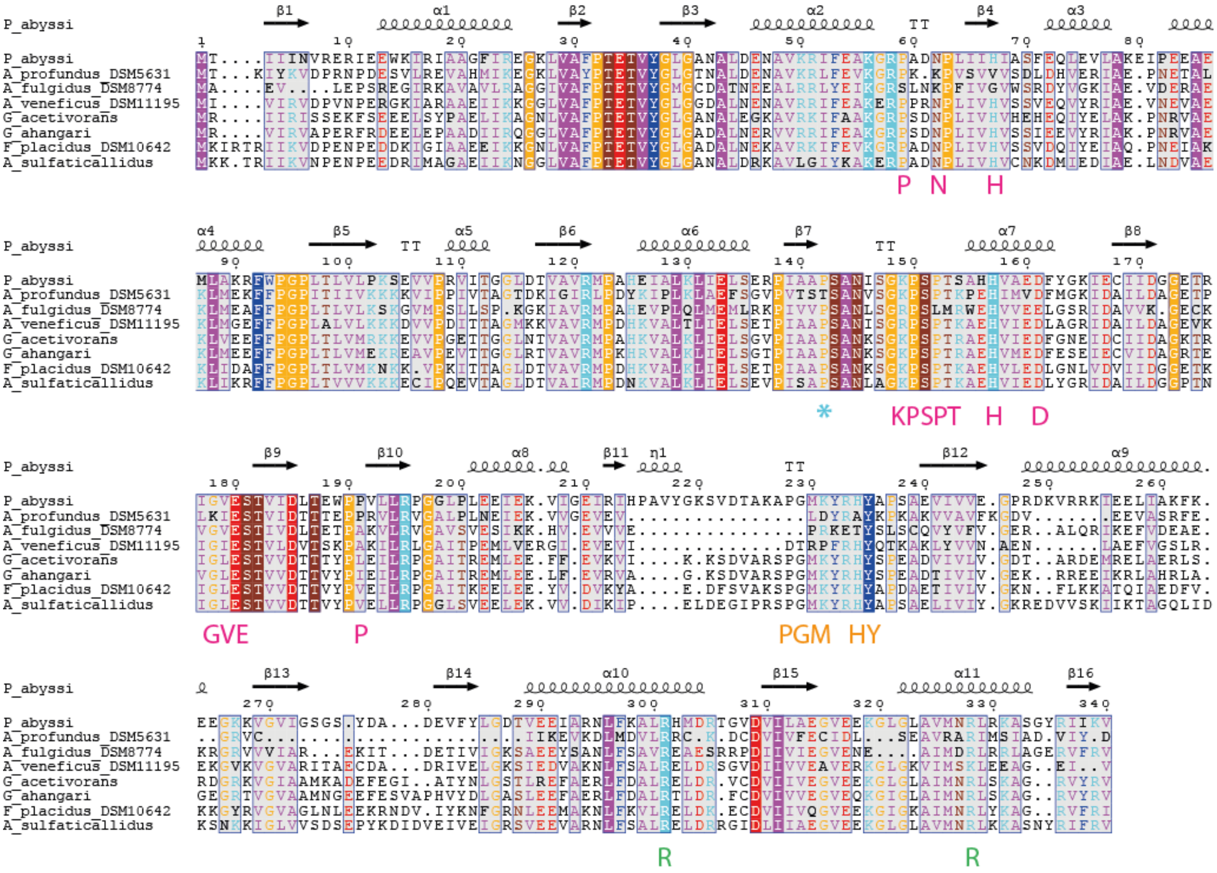


**Supplementary figure 8. The alignment of Sua5 proteins from Archaeoglobi archaea.**

The sequences of Sua5 proteins from seven isolated species were retrieved from Uniprot and aligned using MAFFT. The distribution of the secondary structures, shown on the top of the alignment, was rendered using ESPript3 web server. The structure of the *Pa*-Sua5 was used as reference (PDB: 6F87). The Sua5-specific conserved residues are indicated below the alignment. The residues conserved in the TsaC-like domain are in pink, those found in the interdomain loop are in orange and those found in the SUA5 domain are in green. The signature residue $Pro^{143}$ is highlighted with a cyan asterisk.

## Supplementary table 1. Distribution of Sua5 and TsaC orthologs across the tree of life.

| | | | Group | %Sua5 | Sub-group | Nb of sequences | Nb of Sua5 (PSAN + HY) | %Sua5 | Database update |
|---|---|---|---|---|---|---|---|---|---|
| Bacteria | | B1 | Cyanobacteria | 1% | | 377 | 4 | 1,1% | 2021-06 |
| | | B2 | Thermotogae | 100% | | 57 | 57 | 100% | 2021-06 |
| | | B3 | Deinococcus-Thermus | 22% | Meiothermus | 12 | 12 | 100% | 2021-06 |
| | | | | | Trueperaceae | 2 | 2 | 100% | 2021-06 |
| | | | | | Others | 50 | 0 | 0% | 2021-06 |
| | | B4 | Chloroflexi | 40% | Anaerolineae | 33 | 3 | 9,1% | 2021-06 |
| | | | | | Ardenticatenia | 3 | 1 | 33,3% | 2021-06 |
| | | | | | Caldilineae | 1 | 1 | 100% | 2021-06 |
| | | | | | Candidatus Thermofonsia | 3 | 3 | 100% | 2021-06 |
| | | | | | Chloroflexia | 13 | 11 | 84,6% | 2021-06 |
| | | | | | Dehalococcoidia | 10 | 0 | 0% | 2021-06 |
| | | | | | Ktedonobacteria | 12 | 12 | 100% | 2021-06 |
| | | | | | Thermomicrobia | 3 | 0 | 0% | 2021-06 |
| | | B5 | Actinobacteria | 2% | Acidimicrobia | 17 | 7 | 41,2% | 2021-06 |
| | | | | | Actinomycetia | 1264 | 12 | 0,9% | 2022-11 |
| | | | | | Coriobacteriia | 71 | 0 | 0,0% | 2022-11 |
| | | | | | Nitriliruptoria | 11 | 8 | 72,7% | 2022-11 |
| | | | | | Rubrobacteria | 19 | 0 | 0,0% | 2022-11 |
| | | | | | Thermoleophilia | 34 | 1 | 2,9% | 2022-11 |
| | | | | | Actinobacteria incertae sedis | 13 | 6 | 46,2% | 2022-11 |
| | | B6 | Firmicutes | 31% | Bacilli | 500 | 4 | 0,8% | 2022-11 |
| | | | | | Clostridia | 55 | 26 | 47,3% | 2022-11 |
| | | | | | Erysipelotrichia | 92 | 0 | 0,0% | 2022-11 |
| | | | | | Negativicutes | 192 | 185 | 96,4% | 2022-11 |
| | | | | | Tissierellia | 124 | 124 | 100,0% | 2022-11 |
| | | | | | Firmicutes incertae sedis | 143 | 3 | 2,1% | 2022-11 |
| | | B7 | Spirochaetia | 85% | Brachyspirales | 21 | 0 | 0,0% | 2022-11 |
| | | | | | Leptospirales | 122 | 122 | 100,0% | 2022-11 |
| | | B8 | Bacteroidetes/Chlorobi group | 0% | | 962 | 1 | 0,1% | 2022-11 |
| | | B9 | Planctomycetes | 67% | | 386 | 260 | 67,4% | 2022-11 |
| | | B10 | Verrucomicrobia | 100% | | 381 | 381 | 100,0% | 2022-11 |
| | | B11 | Chlamydiae | 49% | | 70 | 34 | 48,6% | 2022-11 |
| | | B12 | Delta/Epsilon Proteobacteria | 0% | | 143 | 0 | 0,0% | 2022-11 |
| | | B13 | Alpha Proteobacteria | 39% | | 89 | 35 | 39,3% | 2022-11 |
| | | B14 | Beta Proteobacteria | 31% | | 29 | 9 | 31,0% | 2022-11 |
| | | B15 | Gamma Proteobacteria | 0% | | 576 | 0 | 0,0% | 2022-11 |
| Eukaryotes | | E1 | Heterolobosea | 100% | | 1 | 1 | 100% | 2021-06 |
| | | E2 | Cryptophyta | 100% | | 1 | 1 | 100% | 2021-06 |
| | | E3 | Rhodophyta | 75% | | 4 | 3 | 75% | 2021-06 |
| | | E4 | Stramenopiles | 100% | | 36 | 36 | 100% | 2021-06 |
| | | E5 | Alveolata | 100% | | 43 | 43 | 100% | 2021-06 |
| | Viridiplantae | E6 | Chlorophyta | 54% | Chlorophyceae | 7 | 7 | 100% | 2021-06 |
| | | | | | Others | 6 | 0 | 0% | 2021-06 |
| | | E7 | Streptophyta | 0% | | 113 | 0 | 0% | 2021-06 |
| | | E8 | Amoebozoa | 100% | | 7 | 7 | 100% | 2021-06 |
| | Opisthokonta | E9 | Fungi | 100% | | 680 | 678 | 99,7% | 2021-06 |
| | | E10 | Opisthokonta incertae sedis | 50% | | 2 | 1 | 50% | 2021-06 |
| | | E11 | Choanoflagellida | 0% | | 2 | 0 | 0% | 2021-06 |
| | | E12 | Metazoa | 1% | | 422 | 3 | 0,7% | 2021-06 |
| Archaea | | A1 | Thaumarchaea | 8% | | 86 | 7 | 8,1% | 2021-06 |
| | | A2 | Thermoproteales | 93% | | 30 | 28 | 93% | 2021-06 |
| | | A3 | Sulfolobales | 100% | | 17 | 17 | 100,00% | 2021-06 |
| | | A4 | Desulfurococcales | 50% | Desulfurococcaceae | 13 | 9 | 69,2% | 2021-06 |
| | | | | | Pyrodictiaceae | 5 | 0 | 0% | 2021-06 |
| | | A5 | Asgard | 11% | | 9 | 1 | 11% | 2021-06 |
| | | A6 | Thermococci | 100% | | 39 | 39 | 100% | 2021-06 |
| | | A7 | Methanococci | 13% | | 15 | 2 | 13,3% | 2021-06 |
| | | A8 | Methanobacteria | 0% | | 56 | 0 | 0% | 2021-06 |
| | | A9 | Methanomassiliicoccales | 0% | | 11 | 0 | 0% | 2021-06 |
| | | A10 | Thermoplasmatales | 50% | | 28 | 14 | 50% | 2021-06 |
| | | A11 | DHVE2 | 0% | | 2 | 0 | 0% | 2021-06 |
| | | A12 | Archaeoglobi | 89% | | 9 | 8 | 89% | 2021-06 |
| | | A13 | Methanomicrobia | 47% | Methanosarcineae | 52 | 52 | 100% | 2021-06 |
| | | | | | Others | 67 | 4 | 6% | 2021-06 |

NCBI taxonomy database was used to classify organisms into group and subgroup categories. Nb of sequences = number of TsaC and Sua5 sequences that matched filtering criteria (see materials and methods). Nb of Sua5 = number of sequences corresponding uniquely to Sua5 proteins. %Sua5 = percentage of Sua5 sequences in the total number of TsaC and Sua5 sequences.

## Supplementary table 2. Sua5 sequences used for phylogenetic inference

| Sua5 | Species | Uniprot ID |
|---|---|---|
| Archaea | Metallosphaera sedula (strain ATCC 51363 / DSM 5348 / JCM 9185 / NBRC 15509 / TH2) | A4YI99 |
| | Sulfurisphaera tokodaii (strain DSM 16993 / JCM 10545 / NBRC 100140 / 7) (Sulfolobus tokodaii) | Q970S6 |
| | Ferroplasma acidarmanus fer1 | S0ATU2 |
| | Caldivirga maquilingensis (strain ATCC 700844 / DSM 13496 / JCM 10307 / IC-167) | A8MAU3 |
| | Pyrobaculum aerophilum (strain ATCC 51768 / IM2 / DSM 7523 / JCM 9630 / NBRC 100827) | Q8ZU26 |
| | Thermoproteus tenax (strain ATCC 35583 / DSM 2078 / JCM 9277 / NBRC 100435 / Kra 1) | G4RQ02 |
| | Fervidobacterium nodosum (strain ATCC 35602 / DSM 5306 / Rt17-B1) | A7HKY2 |
| | Vulcanisaeta moutnovskia (strain 768-28) | F0QT00 |
| | Methanococcoides burtonii (strain DSM 6242 / NBRC 107633 / OCM 468 / ACE-M) | Q12ZH0 |
| | Archaeoglobus veneficus (strain DSM 11195 / SNP6) | F2KMZ1 |
| | Ferroglobus placidus (strain DSM 10642 / AEDII12DO) | D3RX12 |
| | Methanosarcina mazei C16 | A0A0E3RQV9 |
| | Candidatus Nitrosarchaeum limnium BG20 | S2EJK3 |
| | Pyrococcus abyssi (strain GE5 / Orsay) | Q9UYB2 |
| | Thermococcus barophilus (strain DSM 11836 / MP) | F0LMR7 |
| | Thermococcus nautili | W8PK17 |
| | Desulfurococcus amylolyticus (strain DSM 18924 / JCM 16383 / VKM B-2413 / 1221n) | B8D397 |
| | Thermofilum pendens (strain DSM 2475 / Hrk 5) | A1S178 |
| | Methanocaldococcus infernus (strain DSM 11812 / JCM 15783 / ME) | D5VS69 |
| Bacteria | Truepera radiovictrix (strain DSM 17093 / CIP 108686 / LMG 22925 / RQ-24) | D7CVC7 |
| | Burkholderia ambifaria IOP40-10 | B1F9L4 |
| | Anaerolinea thermophila (strain DSM 14523 / JCM 11388 / NBRC 100420 / UNI-1) | E8MZI9 |
| | Phaeodactylibacter xiamenensis | A0A098SDY5 |
| | Coraliomargarita akajimensis (strain DSM 45221 / IAM 15411 / JCM 23193 / KCTC 12865 / 04OKA010-24) | D5ENH8 |
| | Opitutus terrae (strain DSM 11246 / JCM 15787 / PB90-1) | B1ZTU9 |
| | Acetobacter pasteurianus (strain NBRC 3283 / LMG 1513 / CCTM 1153) | C7JEN7 |
| | Bartonella bacilliformis Ver097 | A0A072REI2 |
| | Dinoroseobacter shibae (strain DSM 16493 / NCIMB 14021 / DFL 12) | A8LL68 |
| | Gemmata sp. SH-PL17 | A0A142X7K1 |
| | Rhodopirellula baltica (strain DSM 10527 / NCIMB 13988 / SH1) | Q7UPX2 |
| | Bacillus subtilis (strain 168) | P39153 |
| | Listeria innocua serovar 6a (strain ATCC BAA-680 / CLIP 11262) | Q927V2 |
| | Natranaerobius thermophilus (strain ATCC BAA-1301 / DSM 18059 / JW/NM-WN-LF) | B2A3I0 |
| | Rhodothermus marinus (strain ATCC 43812 / DSM 4252 / R-10) (Rhodothermus obamensis) | D0MJ17 |
| | Chlorobaculum parvum (strain NCIB 8327) | B3QLK9 |
| | Kosmotoga olearia (strain ATCC BAA-1733 / DSM 21960 / TBF 19.5.1) | C5CGY8 |
| | Thermotoga maritima (strain ATCC 43589 / MSB8 / DSM 3109 / JCM 10099) | Q9WZV6 |
| | Aeropyrum pernix (strain ATCC 700893 / DSM 11879 / JCM 9820 / NBRC 100138 / K1) | Q9Y988 |
| | Legionella longbeachae serogroup 1 (strain NSW150) | D3HMZ0 |
| | Simkania negevensis (strain ATCC VR-1471 / Z) | F8L7W8 |
| | Borrelia afzelii (strain PKo) | Q0SMC2 |
| | Treponema azotonutricium (strain ATCC BAA-888 / DSM 13862 / ZAS-9) | F5YB50 |
| Eukaryotes | Plasmodium falciparum (isolate 3D7) | Q8I610 |
| | Plasmodium vivax | A0A565A063 |
| | Toxoplasma gondii | Q1JT85 |
| | Oxytricha trifallax | J9HLG1 |
| | Dictyostelium purpureum (Slime mold) | F0ZK67 |
| | Naegleria gruberi (Amoeba) | D2UXF9 |
| | Thalassiosira pseudonana (Marine diatom) (Cyclotella nana) | B8C029 |
| | Phytophthora infestans (strain T30-4) (Potato late blight fungus) | D0N186 |
| | Babesia bigemina | A0A061DBE3 |
| | Aureococcus anophagefferens (Harmful bloom alga) | F0YJT4 |
| | Saccharomyces cerevisiae (strain ATCC 204508 / S288c) (Baker's yeast) | P32579 |
| | Yarrowia lipolytica (Candida lipolytica) | A0A371C9H7 |
| | Schizosaccharomyces pombe (strain 972 / ATCC 24843) (Fission yeast) | O94530 |
| | Aspergillus fumigatus Z5 | A0A0J5Q2G4 |
| | Ustilago maydis (strain 521 / FGSC 9021) (Corn smut fungus) | A0A0D1C8D3 |
| | Mortierella elongata AG-77 | A0A197JTS7 |
| | Chlamydomonas reinhardtii (Chlamydomonas smithii) | A0A2K3DSN0 |
| | Volvox carteri f. nagariensis | D8TKZ3 |
| | Batrachochytrium dendrobatidis (strain JAM81 / FGSC 10211) (Frog chytrid fungus) | F4NSM7 |
| | Galdieria sulphuraria (Red alga) | M2WPY0 |
| | Guillardia theta (strain CCMP2712) (Cryptophyte) | L1IT80 |
| | Chondrus crispus (Carrageen Irish moss) (Polymorpha crispa) | S0F320 |
| | Capsaspora owczarzaki (strain ATCC 30864) | A0A0D2WT10 |
| | Acanthamoeba castellanii str. Neff | L8GLU7 |

## Supplementary table 3. TsaC sequences used for phylogenetic inference

| TsaC | Species | Uniprot ID |
|---|---|---|
| Archaea | Nitrososphaera gargensis (strain Ga9.2) | K0IIU5 |
| | Nitrosopumilus maritimus (strain SCM1) | A9A3R1 |
| | Lokiarchaeum sp. (strain GC14_75) | A0A0F8W7Z2 |
| | Caldiarchaeum subterraneum | E6N7J8 |
| | Pyrolobus fumarii (strain DSM 11204 / 1A) | G0EH40 |
| | Candidatus Thorarchaeota archaeon AB_25 | A0A1Q9P493 |
| | Candidatus Odinarchaeota archaeon LCB_4 | A0A1Q9N788 |
| | Methanocella paludicola (strain DSM 17711 / JCM 13418 / NBRC 101707 / SANAE) | D1YXA0 |
| | Candidatus Methanomethylophilus sp. 1R26 | A0A0W7THM7 |
| | Methanosaeta harundinacea (strain 6Ac) | G7WL99 |
| | Natrialba magadii (strain ATCC 43099 / DSM 3394 / CIP 104546 / JCM 8861/ NBRC 102185 / NCIMB 2190 / MS3) | D3SUM8 |
| | Haloarcula marismortui (strain ATCC 43049 / DSM 3752 / JCM 8966 / VKM B-1809) (Halobacterium marismortui) | Q5UZB1 |
| | Methanoregula boonei (strain DSM 21154 / JCM 14090 / 6A8) | A7I9S8 |
| | Methanocorpusculum labreanum (strain ATCC 43576 / DSM 4855 / Z) | A2SSU7 |
| | Methanothermobacter thermautotrophicus (strain ATCC 29096 / DSM 1053 / JCM 10044 / NBRC 100330 / Delta H) | O27727 |
| | Methanothermus fervidus (strain ATCC 43054 / DSM 2088 / JCM 10308 / V24 S) | E3GWF7 |
| | Methanobrevibacter smithii CAG:186 | R7PTK6 |
| | Aciduliprofundum boonei (strain DSM 19572 / T469) | D3TDC9 |
| | Methanotorris igneus (strain DSM 5666 / JCM 11834 / Kol 5) | F6BCB4 |
| | Methanococcus vannielii (strain ATCC 35089 / DSM 1224 / JCM 13029 / OCM 148 / SB) | A6URG4 |
| | Ignicoccus hospitalis (strain KIN4/I / DSM 18386 / JCM 14125) | A8A9F4 |
| Bacteria | Synechocystis PCC 680 slr1265 | P74144 |
| | Anaerolinea thermophila | A0A117LGS8 |
| | Chlamydia muridarum (strain MoPn / Nigg) | Q9PKQ1 |
| | Desulfobulbus propionicus (strain ATCC 33891 / DSM 2032 / 1pr3) | A0A7U3YM60 |
| | Cyanothece sp. CCY0110 | A3IN36 |
| | Brachyspira hyodysenteriae (strain ATCC 49526 / WA1) | A0A3B6VB06 |
| | Pelobacter carbinolicus (strain DSM 2380 / NBRC 103641 / GraBd1) | Q3A498 |
| | Bacteroides fragilis | A0A149N1N2 |
| | Thiobacillus denitrificans (strain ATCC 25259) | Q3SG43 |
| | Escherichia coli (strain K12) | P45748 |
| | Acinetobacter baumannii (strain ACICU) | B2I111 |
| | Thermomicrobium roseum (strain ATCC 27502 / DSM 5159 / P-2) | B9KY83 |
| | Marinithermus hydrothermalis (strain DSM 14884 / JCM 11576 / T1) | F2NL90 |
| | Deinococcus radiodurans (strain ATCC 13939 / DSM 20539 / JCM 16871 / LMG 4051 / NBRC 15346)) | Q9RTA3 |
| | Kineococcus radiotolerans (strain ATCC BAA-149 / DSM 14245 / SRS30216) | A6W7F9 |
| | Streptosporangium roseum (strain ATCC 12428 / DSM 43021 / JCM 3005 / NI 9100) | D2ARX0 |
| | Catenulispora acidiphila (strain DSM 44928 / NRRL B-24433 / NBRC 102108 / JCM 14897) | C7Q635 |
| Eukaryotes | Aspergillus fumigatus Z5 | A0A0J5PIE0 |
| | Amphimedon queenslandica (Sponge) | A0A1X7UH48 |
| | Xenopus tropicalis (Western clawed frog) (Silurana tropicalis) | Q5FVA3 |
| | Homo sapiens (Human) | Q86U90 |
| | Mus musculus (Mouse) | Q3U5F4 |
| | Monosiga brevicollis (Choanoflagellate) | A9V2X9 |
| | Drosophila melanogaster (Fruit fly) | Q8SYJ9 |
| | Salpingoeca rosetta (strain ATCC 50818 / BSB-021) | F2UHZ0 |
| | Ostreococcus lucimarinus (strain CCE9901) | A4RRF7 |
| | Micromonas commoda (strain RCC299 / NOUM17 / CCMP2709) (Picoplanktonic green alga) | C1FE24 |
| | Selaginella moellendorffii (Spikemoss) | D8SHM4 |
| | Physcomitrella patens subsp. patens (Moss) | A0A2K1LB18 |
| | Oryza sativa subsp. indica (Rice) | A2XDM4 |
| | Arabidopsis thaliana (Mouse-ear cress) | F4K0E6 |
